# Inter-genome comparison of the Quorn fungus *Fusarium venenatum* and the closely related plant infecting pathogen *Fusarium graminearum*

**DOI:** 10.1186/s12864-018-4612-2

**Published:** 2018-04-19

**Authors:** Robert King, Neil Andrew Brown, Martin Urban, Kim E. Hammond-Kosack

**Affiliations:** 1 Department of Computational and Analytical Sciences, Rothamsted Research, Harpenden, Herts AL5 2JQ UK; 2 Department of Biointeractions and Crop Protection, Rothamsted Research, Harpenden, Herts AL5 2JQ UK; 3 Department of Biology & Biochemistry, University of Bath, Claverton Down, Bath, BA2 7AY UK

**Keywords:** *Fusarium graminearum*, *Fusarium venenatum*, Comparative genome analyses, Secondary metabolite clusters, Secretome, PHI-base genes, Species-specific genes

## Abstract

**Background:**

The soil dwelling saprotrophic non-pathogenic fungus *Fusarium venenatum*, routinely used in the commercial fermentation industry, is phylogenetically closely related to the globally important cereal and non-cereal infecting pathogen *F. graminearum*. This study aimed to sequence, assemble and annotate the *F. venenatum* (strain A3/5) genome, and compare this genome with *F. graminearum*.

**Results:**

Using shotgun sequencing, a 38,660,329 bp *F. venenatum* genome was assembled into four chromosomes, and a 78,618 bp mitochondrial genome. In comparison to *F. graminearum*, the predicted gene count of 13,946 was slightly lower. The *F. venenatum* centromeres were found to be 25% smaller compared to *F. graminearum*. Chromosome length was 2.8% greater in *F. venenatum,* primarily due to an increased abundance of repetitive elements and transposons, but not transposon diversity. On chromosome 3 a major sequence rearrangement was found, but its overall gene content was relatively unchanged. Unlike homothallic *F. graminearum*, heterothallic *F. venenatum* possessed the *MAT1–1* type locus, but lacked the *MAT1–2* locus. The *F. venenatum* genome has the type A trichothecene mycotoxin *TRI5* cluster, whereas *F. graminearum* has type B. From the *F. venenatum* gene set, 786 predicted proteins were species-specific versus NCBI. The annotated *F. venenatum* genome was predicted to possess more genes coding for hydrolytic enzymes and species*-*specific genes involved in the breakdown of polysaccharides than *F. graminearum*. Comparison of the two genomes reduced the previously defined *F. graminearum-*specific gene set from 741 to 692 genes. A comparison of the *F. graminearum* versus *F. venenatum* proteomes identified 15 putative secondary metabolite gene clusters (SMC), 109 secreted proteins and 38 candidate effectors not found in *F. venenatum*. Five of the 15 *F. graminearum-specific* SMCs that were either absent or highly divergent in the *F. venenatum* genome showed increased in planta expression. In addition, two predicted *F. graminearum* transcription factors previously shown to be required for fungal virulence on wheat plants were absent or exhibited high sequence divergence.

**Conclusions:**

This study identifies differences between the *F. venenatum* and *F. graminearum* genomes that may contribute to contrasting lifestyles, and highlights the repertoire of *F. graminearum*-specific candidate genes and SMCs potentially required for pathogenesis.

**Electronic supplementary material:**

The online version of this article (10.1186/s12864-018-4612-2) contains supplementary material, which is available to authorized users.

## Background

The genus *Fusarium* contains numerous destructive and toxigenic filamentous ascomycete fungi that pose a current, and growing threat, to plant, animal, human and ecosystem health. Prominent plant diseases caused by *Fusaria* include, *Fusarium* head blight (FHB) of cereals [1, 2], sudden death syndrome of soybeans [3], and vascular wilts of numerous important crops, such as banana, tomato and oil palm [4]. The negative impact of *Fusarium*-induced yield losses is compounded by the contamination of the crop with various mycotoxins, making the harvest unsuitable for human or animal consumption. Additionally, *Fusaria* can cause opportunistic, and life-threatening, infections of immunocompromised humans [5]. Other *Fusaria* are non-pathogenic and have a saprophytic or endophytic lifestyle [6]. The close phylogenetic relationships but diverse array of fungal lifestyles present within *Fusaria* are ideal for the identification of the genetic factors contributing to the occupancy of various biological and environmental niches, and the evolution of different lifestyles.

Fusarium head blight disease, also known as Fusarium head scab in the United States and elsewhere, is caused by up to 17 related Fusarium species and occurs on all small grain cereals throughout the globe. Within Europe, the USA, China, Brazil and elsewhere, the main causal organism on wheat, barley and maize crops is *F. graminearum.* FHB is the number one floral disease affecting wheat crops and is a serious health hazard, due to direct crop losses and the contamination of the grain with type B trichothecene mycotoxins, such as deoxynivalenol (DON) and nivalenol [1, 2]. Altered agronomic practices have failed to reduce inoculum levels, while fungicides only provide modest crop protection, and fully FHB-resistant wheat and barley cultivars have not been realised. Consequently, new approaches to control FHB are needed to improve grain yield, quality and safety.

At present, *F. graminearum* is known to secrete a cocktail of enzymatic and non-enzymatic proteinaceous virulence factors in combination with host and non-host specific, toxic and non-toxic, metabolites into the plant tissue to manipulate the host and to obtain nutrition [7]. The independent loss of the secretion of i) the DON mycotoxin [8], ii) the iron scavenging triacetylfusarinine C (TAFC) siderophore [9], or iii) the Fgl1 lipase [10], results in a dramatic reduction in virulence, demonstrating that each secreted biologically active compound is required for full infection. Other unknown fungal compounds, metabolites and proteins potentially contribute to the establishment of infection and disease progression. Recent advancements have improved the understanding of the infection biology of *F. graminearum* in wheat [11, 12], fungal virulence factor requirements [8–10, 13] and some of the underlying plant resistance mechanisms [14]. However, an increased understanding of the evolution of *F. graminearum* as a pathogen of cereals may accelerate the rate of discovery of additional virulence determinants, and assist in the development of novel crop protection strategies.

*Fusarium venenatum* A3/5 is a ubiquitous saprophytic, soil-dwelling fungus, which was initially misclassified as *Fusarium graminearum* A3/5, prior to the utilisation of molecular phylogenetic techniques [15]. In the 1980’s, *F. venenatum* was developed as a protein-rich alternative to meat, which was low in fat and high in dietary fibre, and subsequently commercialised under the name Quorn® [16–18]. The production of Quorn, involves fungal biomass accumulation in fermenters under specific growth conditions, which is then heated, mixed with egg albumin or potato starch, and rolled into meat-like fibres. To date, Quorn remains the only fungal-derived protein source (myco-protein) that is commercially available and approved for human consumption, throughout the world. Industrially, *F. venenatum* has also been widely used as a biological system for the production of recombinant proteins, including trypsin, lipases, phytases and xylanases [19–22]. Therefore, the saprophyte *F. venenatum* has become an economically important microbial cell factory for the production of myco-protein and either industrial or food grade enzymes. No detectable toxins are found in any of the fermentation products. However, *F. venenatum* has been shown to produce the type A trichothecene, diacetoxyscirpenol (DAS) toxin in inoculated rice grain cultures [15].

Single species genomics when combined with comparative genomics is a powerful tool to study how fungi have adapted to occupy their environmental niche(s), through the acquisition, loss, or diversification of annotated/unannotated protein families and/or chromosomal regions. Sequencing of the *F. graminearum* genome [23] revealed few repetitive sequences, due to an active repeat-induced point mutation mechanism, and polymorphic regions located near telomeres and at several other genome locations which were proposed to represent ancient chromosomal fusion events. These polymorphic regions were found to be enriched for genes, either highly expressed in planta, predicted to code for secreted proteins, or genes confirmed to be involved in pathogenic interactions with plants [23]. Subsequently, the comparison of the genomes from *F. graminearum* with *Fusarium oxysporum* f. sp. *lycopersici* (*Fol*), a pathogen of tomato, and *Fusarium verticillioides*, a pathogen of maize, revealed the existence of small lineage-specific chromosomes in *Fol,* which conferred pathogenicity [24]. This demonstrates the value of comparative genomics to study the evolution of pathogenesis and for the discovery of novel virulence determinants. The recent completion of the *F. graminearum* genome from telomere-to-telomere now provides an excellent resource for further interspecies comparisons [25].

In this study, we present a genomic comparison of the closely related non-pathogenic and pathogenic *Fusaria*, respectively *F. venenatum* and *F. graminearum*. Our analyses revealed the striking genetic and genomic similarities between the two species and have revealed the minimal gene set specific to the pathogenic lifestyle of *F. graminearum*. The predicted number of *F. venentaum* and *F. graminearum* gene models, hereafter referred to as genes (13,946 vs 14,164), and the GC content (47.6 vs 48) were very similar between species. Similarly, the macro- and microsynteny between the two genomes was very high, except for a region on chromosome 3 where a large rearrangement and reorientation was found. A similar genome rearrangement was also found in the closely related species, *F. poae* [26]. The genomic comparison revealed the trichothecene mycotoxin *TRI5* gene cluster resides at the same location on chromosome 2 in both species with *F. venenatum* possessing the type A and *F. graminearum* the type B. The *TRI1/TRI16* cluster is located at the same location on chromosome 1 in both species, but only *F. venenatum* is predicted to possess a functional copy of TRI16. The *F. venenatum* genome was predicted to possess a greater number of genes coding for hydrolytic enzymes and species*-*specific genes involved in the breakdown of polysaccharides in a pair-wise analysis. Both attributes would potentially facilitate a solely saprophytic lifestyle in soil and within an industrial fermenter

Further analysis reduced the previously defined *F. graminearum-*specific gene set [25] from 741 to 692 genes, with the additional comparisons to *F. culmorum* and *F. poae* reducing this number further to 690. Of these 690, there are five secreted proteins with no known annotation and three candidate effectors. A comparison of the proteomes of *F. graminearum* vs *F. venenatum* identified 15 putative secondary metabolite gene clusters, 109 secreted proteins, 38 candidate effectors not found in *F. venenatum*. Exploring the genome for *F. graminearum* homologues of 160 genes proven to be required for virulence using the pathogen-host interactions database (PHI-base) [27, 28], revealed only two genes annotated as transcription factors with a reduced virulence phenotype were absent in *F. venenatum* versus *F. graminearum.* There was no difference in genes annotated with loss of pathogenicity. This genomic comparison of closely related pathogenic and non-pathogenic *Fusarium* species highlights the repertoire of *F. graminearum* specific candidate genes and secondary metabolite clusters potentially required for pathogenesis.

## Results

### Assessment of *Fusarium venenatum* pathogenicity on wheat and tomato

To confirm that *F. venenatum* was not pathogenic on wheat, in contrast to *F. graminearum*, macrospores of both species were generated on potato dextrose agar plates. Wheat heads were drop- and spray-inoculated at anthesis. Disease development and grain formation were assessed at 16 and 21 days post infection (see Additional file 1). In these tests, *F. venenatum* was not able to cause visible disease symptoms on wheat heads, while *F. graminearum* caused significant wheat head bleaching and the abortion of grain development. A very small amount of *F. venenatum* hyphal growth was evident solely on the wheat anthers 2–3 days post inoculation, but this growth did not persist.

To assess whether *F. venenatum* could cause a post-harvest disease on tomato (*Solanum lycopersicum*), ripe fruits were ‘pin-prick’ wounded and inoculated with either *F. venenatum* or *F. graminearum* conidia. In this bioassay, *F. graminearum* rapidly colonised the tomato pericarp within 4 days and developed an abundance of extruding, dry, aerial mycelia. In contrast, *F. venenatum* showed limited ability to colonise tomato fruits and visible ‘water soaked’ mycelia could only be observed after a prolonged 12-day incubation. However, *F. venenatum*, was fully able to proliferate on plant-derived nutrient sources contained within agar media, such as carrot and potato dextrose agar. Both bioassays revealed that *F. venenatum* has a very limited ability to colonise living plant tissues compared to *F. graminearum.*

These in planta results raised the possibility that *F. venenatum* could be used as a biocontrol agent to inhibit growth of pathogenic *Fusarium* species. This hypothesis was tested in a series of co-cultivation experiments. First *F. venenatum* was co-inoculated on carrot agar plates with three wheat-pathogenic *Fusarium* strains, FgPH-1, Fg602 and FcUK99. A zone of vegetative incompatibility [29] was observed between all isolates (see Additional file 2). The extent of the zone of inhibition for *F. venenatum* was no different to the zones observed between other *F. graminearum* strains. Next flowering wheat heads were co-inoculated with *F. venenatum* and *F. culmorum* conidia. The *F. culmorum* UK99 strain was chosen as the pathogen in the co-inoculation experiments, because both FcUK99 and the sequenced *F. venenatum* strain had originally been isolated in the United Kingdom and at the time of isolation *F. culmorum* was the sole FHB causing species in UK wheat crops. Three types of in planta experiments were done. Firstly, *F. culmorum* and *F. venenatum* conidia where mixed in equal amounts and co-inoculated into two spikelets at anthesis. Secondly, *F. venenatum* conidia were sprayed onto wheat heads at the boot stage to potentially prime plant defence responses, followed by point-inoculation of two spikelets per head with *F. culmorum* conidia at anthesis. Thirdly, *F. culmorum* was point-inoculated at anthesis and immediately sprayed a second time with *F. venenatum* conidia. Disease progress was monitored over 20 days. No inhibition of FHB disease development was observed in these co-inoculation experiments (see Additional file 2). Therefore, we conclude that *F. venenatum* is not a biocontrol agent for FHB disease caused by *F. culmorum*.

### Assembly and comparative genomics of the *Fusarium venenatum* genome

An Illumina 100 bp pair-end read approach was taken to sequence the genome. De novo assembly of the *F. venenatum* genome from telomere-to-telomere, with 137× coverage, resulted in a 38,660,329 bp genome, which assembled into four chromosomes, and a 78,618 bp mitochondrial genome. At present 37 gaps remain within the genome, while one supercontig of 9545 bp was unplaced which contained three genes consisting of a transcription factor (FVRRES_13944), a cholinesterase (FVRRES_13945), and a negative transcriptional regulator (FVRRES_13946), These three genes were not found in *F. graminearum* but were found in *F. oxysporum*. This is also the situation for many of the other genes specific to *F. venenatum* vs *F. graminearum* where a BLASTP hit was found in *F. oxysporum.* The GC content was 47.6%, which is comparable to 48% for *F. graminearum* [25]. Gene modelling was performed using Maker2 [30], yielding 13,946 genes. Average gene coding length in *F. venenatum* and *F. graminearum* was respectively 1388 vs. 1372 bp, with 2.78 vs. 2.76 exons per gene (average exon and intron length 500, 497 and 71, 74 bp, respectively). The *F. venenatum* genome sequence and annotation (FV1) has been deposited in the European Nucleotide Archive under accession PRJEB7533 (Table 1).

**Table 1:** Basic statistics of the *F. venenatum* and *F. graminearum* genomes

	*F. venenatum*	*F. graminearum*
Genome size (bp)^a^	38,660,329	38,060,440
Scaffolds^b^	5	5
GC (%) content^c^	47.7 (47.6^d^)	48.2 (48.0^d^)
Spanned gaps	37	1
Predicted genes	13,946	14,164
Average gene length (bp)	1388	1372
Average exon per gene	2.78	2.76
Average exon length (bp)	500	497
Average intron length (bp)	71	74
Repetitive (%)^d^	1.18	1.03
Transposable elements (%)^d^	0.54	0.29
BUSCO^e^	C:98%, F:1.2%, M:0%	C:98%, F:1.5%, M:0%
Average centromere length (range) kbp	45 (40–52)	60 (56–65)
ENA project accession	PRJEB7533	PRJEB5475

^a^including all scaffolds, N bases, and the mitochondria

^b^including all scaffolds, N bases excluding the mitochondria

^c^excluding N’s and mitochondria

^d^excluding N’s, mitochondria and large repetitive sequence at the carboxyl end of chromosome 4

^e^1438 core fungal genes from BUSCO, C = complete single copy, F = fragmented, M = missing

Previous phylogenetic analyses based on the RNA polymerases, *RPB1* and *RPB2*, concluded that *F. venenatum* was situated within the trichothecene type A/B clade, which predated the separation of the type A clade containing *F. sporotrichioides* and the type B clade containing *F. graminearum* and *F. pseudograminearum* [31]*.* Our analysis confirmed these previous findings but involved a larger cohort of genes. BUSCO was used to identify 904 common proteins however due to a lack of an available genome for *F. sporotrichioides,* this species was replaced with another type A producer, *F. langsethiae* (see Additional files 3 and 4). BLASTP top hits of *F. venenatum* to NCBI identified the greatest number of gene similarities with *F. pseudograminearum*, see Additional file 5, which is a type B trichothecene producer whereas *F. venenatum* has previously been shown to be capable of producing the type A trichothecene, (DAS) under specific cultural conditions [15]. The 904 BUSCO common genes did not include the *TRI* cluster genes and previous research findings from Ward et al. suggested the *TRI* clusters evolved independently from the rest of the genome [32]. Therefore although using non-*TRI* cluster genes to designate type A/B may not be considered applicable, our results match those reported by O’Donnell et al. [31].

**Fig. 1:**
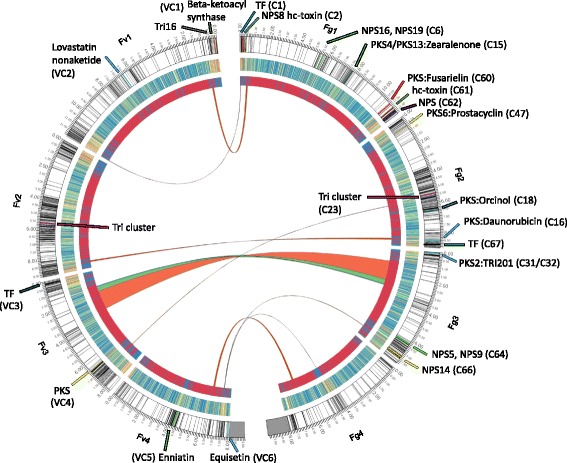
Circos plot of genome comparison between *F. venenatum* and *F. graminearum*. The four chromosomes of each species are represented in a mirror image with the predicted secretomes highlighted in black on the ideograms. The secondary metabolite clusters unique to each species are coloured on the ideograms and labelled “C” for Fg or “VC” for Fv, with prominent gene functions where applicable. The *TRI* cluster is the exception being found in both species but is represented on the plot due to the importance of trichothecene mycotoxin production to virulence in Fg. *TRI*16 is also labelled on Fv because this *TRI* gene is not present in *F. graminearum* PH-1 and is not found in the TRI cluster. The second ring is a heatmap of the protein similarity between the two species with blue representing high and red low similarity. The third ring is a red and blue representation of the similarity from a genome wide alignment with red regions representing an alignment and blue none. Both the low similarity regions from the protein annotation blast comparisons and a lastz alignment of the genome show the regions with a high rate of recombination, such as the secretome regions, and are therefore less similar to one another. The links between the two species in the form of ribbons show translocations and inversions of regions between the two genomes. The large inversion on chromosome 3 has three parts coloured due to translocations within this region and Additional file 9 provides a detailed view of this region

Genome completeness, genome length, number of chromosomes, centromere position and GC content, were all very similar for *F. venenatum* and the closely related pathogen, *F. graminearum* (Table 1). In addition, considerable synteny was found between the genes predicted to reside either side of the four centromeres predicted for each species (see Additional file 6). However, the centromeres were found to be 25% smaller (Table 1 and Additional file 6). Inter-comparison of the chromosome lengths revealed chromosome length was 2.8% larger in *F. venenatum,* with chromosome 3 showing the greatest length increase at 7% (Additional file 7). This length increase was mainly caused by an increased presence of repetitive elements and transposon sequences (described in greater detail below). The *F. venenatum* and *F. graminearum* genomes also had a comparable set of RNAs (see Additional file 8). The synteny between the *F. venenatum* and *F. graminearum* genomes was very high, except for a region on chromosome 3 where three large rearrangements and reorientations were identified within *F. graminearum* chromosome 3 at positions 1036–2645, 2645–3046 and 3046–3112 kbp (Fig. 1 and Additional file 9)*.* This rearrangement was also found in the closely related species, *F. poae* (see Additional file 9). No genes have been disrupted in the rearrangement between *F. venenatum* and *F. graminearum*. Of the 783 annotated genes in v4.0 and 786 in v5.0 present within this chromosomal region in *F. graminearum* (1036–3112 kbp) (Additional file 9), one secondary metabolite cluster (C33) which produces the metabolite ferricrocin [33] is found but this cluster is also present in *F. venenatum* and *F. poae* (BLASTP). The presence of this rearrangement in a closely related pathogenic species *F. poae*, suggests this rearrangement predates the speciation of *F. poae* and *F. venenatum*, and its presence per se is not a likely causal reason for *F. venenatum* loss of virulence.

The quantity but not diversity of repetitive elements including transposon sequences (see Additional file 10) were higher in *F. venenatum* in comparison to *F. graminearum* (1.18% total, 0.54% transposon vs 1.03% and 0.29%) and accounts for most of the increase in chromosome length previously described. *F. graminearum* has been experimentally proven to have an active repeat induced point mutation (RIP) defence mechanism [23]*.* The presence of the same mechanism in *F. venenatum* of gene inactivation is partially supported by the presence of an orthologue of the DNA methyltransferase RID (RIP defective) required for RIP in *N. crassa* [34, 35] and the orthologous neighbouring 10 genes.

The recent evolution of *F. graminearum* in the homothallic FGSG species complex, means the fungus can produce fruiting bodies in the absence of a compatible partner, and the genome contains functional copies of both the *MAT1–1* and *MAT1–2* mating loci [23, 31]. The genome of *F. venenatum* A3/5 strain was found only to possess the MAT1–1 type locus and is likely a heterothallic species (see Additional file 11). However, a sexual stage has not been reported [36]. This requirement for a compatible sexual partner may reduce the frequency of sexual recombination.

These analyses reveal that the overall genome structure of *F. venenatum* and *F. graminearum* were very similar, despite their distinct saprophytic and dual saprophytic/pathogenic lifestyles, respectively. Consequently, subtle differences in the evolution of the closely related fungi and their genomes, may influence the outcome of their various interactions with other organisms and/or the environment.

### Comparison of *F. venenatum* and *F. graminearum* genome annotations

To facilitate interspecies comparisons, the *F. venenatum* genome was annotated using the identical Maker pipeline with the same gene predictors previously used for the completed *F. graminearum* genome [25] thereby generating highly comparable datasets. The exception is that no *F. venenatum* RNA-seq data was available for use. Instead *F. graminearum* RNA-seq data was combined with *Fusarium* proteome evidence to further improve gene prediction. A global BLASTP analysis of the predicted *F. venenatum* proteome at 10^− 5^ and at 10^− 20^ E-value cutoffs to the National Center for Biotechnology Information (NCBI) nr database (1st March 2017) found 786 (99% no BLASTP annotation) and 1149 (94% no BLASTP annotation) unique genes, respectively (see Additional file 12). These *F. venenatum* species-specific genes in relation to NCBI database contents, are found distributed across all four chromosomes (see Additional file 13).

**Fig. 2:**
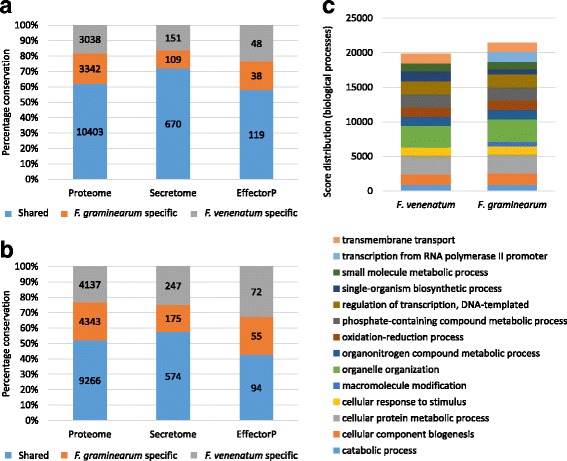
Conservation of the fungal proteome, secretome and putative effectors among *Fusarium venenatum* and *F. graminearum.* The percentage conservation and the number of conserved, or species-specific proteins, based on either 50% (**a**) or 70% (**b**) target and query alignment coverage is presented. Annotation of *Fusarium venenatum* and *F. graminearum* proteomes (**c**) reveals strikingly similar functional profiles

**Fig. 3:**
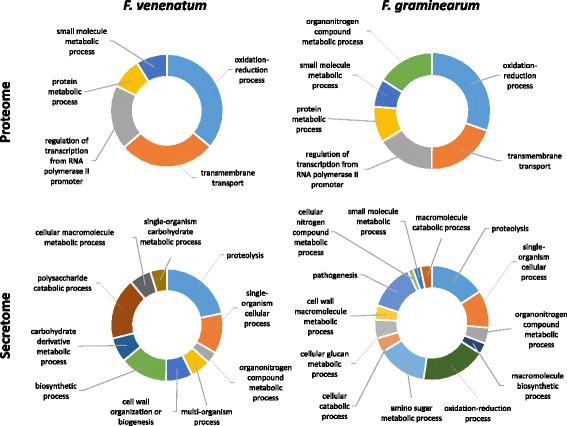
The functional annotation of the *Fusarium venenatum* and *F. graminearum* species-specific proteome and secretome. The proportional representation of the gene ontologies (biological processes) assigned to the species-specific proteins is presented. Analysis based on a 70% target and query alignment and the score distribution

To explore the *F. venenatum* genome in greater detail, a comparison of the predicted proteomes of *F. venenatum* and *F. graminearum* (BLASTP of proteomes using a cutoff of 50% or 70% coverage from a global alignment for both the query and target) revealed that at 70% coverage, ~ 50% of the proteome was conserved between the two *Fusaria*, whilst the remainder was identified to be potentially specific to each species or be highly divergent (Fig. 2, Additional file 14). These species-specific sub-proteomes of the two *Fusaria* predominantly depicted identical functional profiles predicted by gene ontology (biological process annotations) with the exception for *F. graminearum* encoding proteins involved in orangonitrogen compound metabolic processes, demonstrating as suggested above, a divergence of functionality within these specific functional classes (Fig. 3).

A search for paralogue protein groups (OrthoMCL) confirmed the previous finding that *F. graminearum* lacks any identifiable paralogues. However, 12 paralogue protein groups (each comprising either two or three proteins), were found in *F. venenatum* (see Additional file 15). Only one group (group 12) had any known annotation consisting of a basic-leucine zipper transcription factor, while group 2 and 9 contained predicted secreted candidate effector proteins. There are both adjacent gene (neighbours) paralogue groups, near neighbours (within 2–31 genes) and non-neighbour sets (> 100 genes) representing both recent and distant duplication events.

### Comparisons with other pathogenic *Fusaria* and key fungal species

The annotated *F. venenatum* genome was compared to *F. graminearum* and seven other ascomycetes with highly contrasting lifestyles using protein domain signatures known to be functionally linked to virulence [37] within the PHI-base gene set. The additional species selected were the floral maize pathogen *F. verticillioides*, the floral cereal pathogen *F. langsethiae*, the tomato root and vascular pathogen *F. oxysporum* f. sp. *lycopersici*, the foliar rice pathogen *Magnaporthe oryzae*, the floral rice pathogen *Ustilaginoidea virens*, the saprophytic model filamentous ascomycete *Neurospora crassa* and the soil dwelling biocontrol fungus *Trichoderma virens.* The *F. venenatum* genome revealed a predicted functional profile with striking similarity to the floral cereal pathogens *F. graminearum, F. verticillioides* and *F. langsethiae* (Table 2)*.* This included hundreds of glycoside hydrolases, pectin lyases and peptidase involved in the deconstruction of plant cell components, plus hundreds of cytochrome P450 involved in secondary metabolism and major facilitator superfamily or ABC-like transporters. The hemibiotrophic foliar rice pathogen *M. oryzae* also demonstrated a similar functional profile, while presenting diminished pectinolytic potential and a reduced number of putative transporters. The presence of multiple killer toxin homologues was specific to the three cereal infecting *Fusaria.* Direct comparisons with the model saprophytic fungus *N. crassa*, revealed *F. venenatum* to possess a greater hydrolytic potential, an increased capacity to produce secondary metabolites and a far greater number of putative transporters. One possible explanation for these differences is that the saprophytic fungus *N. crassa* feeds on cellulose and hemicellulose substrates and occupies a very restricted range of plant associated niches in natural ecosystems. Whereas the other *Fusaria* species invade and extract nutrients from living and dead plant tissue, whilst also having the ability to grow and survive in the soil and thereby occupy a wider range of biological and environmental niches. Possibly, wider niche occupancy by *Fusaria* and *T. virens* accounts for the greater number of predicted secondary metabolite clusters and putative transporters in their genomes. Overall, the global functional profile of the annotated genome of the saprophyte *F. venenatum* showed greater similarity to the closely related pathogenic *Fusaria* than other ascomycete saprophytes.

**Table 2:** InterPro domains between *F. venenatum, F. graminearum,* and other fungi with plant pathogenic and/or saprophytic lifestyles. The number of proteins identified with a specific protein domain associated with InterPro ID’s that are linked to fungal pathogenesis are presented

Parent inter ID	Child interpro ID	Annotation	*F. venenatum*	*F. graminearum*	*F. verticillioides*	*F. oxysporum lycopersici*	*M. oryzae*	*U. virens*	*N. crassa* ^a^	*T. virens*	*F. langsethiae*
IPR017853	Glycoside hydrolase superfamily		138	122	150	176	116	53	80	142	112
IPR029058	Alpha/Beta hydrolase fold		303	286	315	393	242	100	132	298	269
	IPR000675	cutinase	13	13	12	13	17	4	3	6	16
	IPR001031	thioesterase	5	6	3	3	6	2	1	4	3
	IPR000383	Xaa-Pro dipeptidyl-peptidase-like domain	3	4	11	11	3	0	1	3	7
	IPR000073	Alpha/beta hydrolase fold-1	72	60	64	82	67	12	26	91	60
	IPR001375	Peptidase S9, prolyl oligopeptidase, catalytic domain	10	9	12	11	6	3	2	7	8
	IPR002018	Carboxylesterase, type B	30	26	27	39	20	5	8	28	25
	IPR002921	Fungal lipase-like domain	8	8	6	9	7	4	5	5	7
	IPR002925	Dienelactone hydrolase	8	8	13	12	7	3	6	14	9
	IPR003140	Phospholipase/carboxylesterase/thioesterase	5	5	4	5	3	3	1	5	5
	IPR013094	Alpha/beta hydrolase fold-3	30	29	45	55	19	10	11	26	25
	IPR029059	Alpha/beta hydrolase fold-5	31	27	33	43	10	4	9	0	0
IPR011050	Pectin lyase fold/virulence factor		40	33	42	48	13	11	12	20	29
	IPR000070	Pectinesterase, catalytic	3	3	3	4	1	2	1	2	3
IPR009003	Peptidase cysteine/serine, trypsin-like		7	8	10	10	4	3	3	6	9
IPR021109	Peptidase aspartic		23	22	25	26	24	17	19	25	23
IPR010829	Cerato-platanin		5	4	4	5	1	1	2	6	4
IPR016161	Aldehyde/histidinol dehydrogenase		35	33	47	53	20	15	15	32	29
IPR015500	Peptidase S8, subtilisin-related		23	27	22	32	25	7	7	30	15
IPR004835	Chitin synthase		11	13	14	13	7	8	7	10	10
IPR001138	Zn(2)-C6 fungal-type DNA-binding domain		398	358	246	681	174	49	127	334	261
IPR001128	Cytochrome P450		116	114	132	160	137	35	44	124	120
IPR011701	Major facilitator superfamily		277	250	311	405	180	63	109	248	220
IPR003439	ABC transporter-like		66	62	71	78	50	37	35	63	63
IPR011009	Protein kinases		174	182	192	242	164	149	141	199	184
IPR015433	Phosphatidylinositol kinase		3	3	3	3	3	3	3	3	3
IPR001283/IPR014044	Cysteine-rich secretory protein, allergen V5/Tpx-1-related/CAP domain		5	5	6	8	6	2	3	3	5
IPR011329	Killer toxin		3	4	6	0	0	0	0	3	0

^a^Ensembl fungi version GCA_000786625

**Fig. 4:**
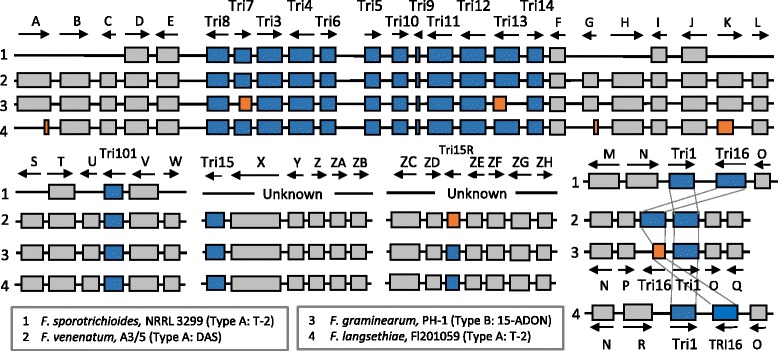
The trichothecene biosynthetic gene clusters within *F. venenatum*, *F. graminearum, F. sporotrichioides* and *F. langsethiae.* Presented is the presence (blue) or absence/loss of function (orange) *TRI* genes within the respective *Fusaria*, in addition to the conservation of the flanking genes beyond the *TRI* clusters (grey). 5′ gene key: A. Haloacid dehydrogenase, B. Glycosyl hydrolase family 115, C. Glycoside hydrolase family 17, D. SGNH hydrolase-type esterase, E. Tyrosinase, M. Membrane protein, N. Gal4-like transcription factor, P. Sugar transporter, R. Glycoside hydrolase, family 29, S. Acyl-CoA N-acyltransferase, T. Phosphate permease, U. Unknown, ZC. Signal transduction histidine kinase, ZD. Isoprenylcysteine carboxyl methyltransferase. 3′ gene key: F. NodB-like polysaccharide deacetylase, G. Unknown, H. Signal peptide containing protein, I. 3-hydroxyacyl-CoA dehydrogenase, J. NADH:cytochrome b5 reductase, K. Unknown, L. Cytochrome P450, E-class, group IV, O. Unknown, Q. WW domain-containing oxidoreductase, V. CTP synthase, W. ATP-citrate lyase/succinyl-CoA ligase, X. Acetyl-CoA synthetase-like, Y. Acyl-CoA N-acyltransferase (siderophore biosynthesis protein), Z. ABC transporter type 1, ZA. Major facilitator transporter, ZB. Major facilitator transporter, ZE. Beta-lactamase/transpeptidase-like, ZF. Peptidase C45, ZG. Thiamin pyrophosphokinase, ZH. Unknown

In a second analysis, the secondary metabolite clusters were predicted for *F. venenatum* and inter-compared with *F. graminearum*. In *F. graminearum* toxic and non-toxic secondary metabolites, such as mycotoxins and siderophores, are essential for virulence against wheat [7–9]. Both *F. venenatum* and *F. graminearum* possess in the same location on chromosome 2 the main *TRI5* cluster responsible for trichothecene mycotoxin biosynthesis and in the same location on chromosome 1 the *TRI1/TRI16* cluster (Fig. 4, Additional file 16). Surrounding both of these *TRI* clusters, the identical gene order (microsynteny) has been maintained. In addition, *F. venenatum* possesses the *TRI101* and *TRI15* genes in the same chromosome location and gene context as in *F. graminearum* (on chromosomes 3 and 4, respectively)*.* Whereas, only a truncated version of the *TRI15R* gene is present in *F. venenatum* at the same location and same gene context on chromosome 1 as in *F. graminearum* (FGRRES_02451) and *F. langsethiae* (FLAG1_06027). A copy of the *TRI201* gene is located on chromosome 3 in *F. venenatum* and chromosome 4 in *F. graminearum* (Additional file 16)*.* Further inspection of the *TRI1/TRI16* cluster revealed an inversion in the relative positions of both genes in these two species compared to the order present in *F. sporotrichioides* and *F. langsethiae.* In *F. graminearum TRI16* is predicted to be truncated. *F. venenatum* is known to be able to produce DAS, a type A trichothecene, that originates from the same biosynthetic pathway as another type A trichothecene, T-2, which is produced by the cereal pathogen *F. sporotrichioides*. This is in contrast to the sequenced *F. graminearum* PH-1 strain that produces the type B trichothecenes DON [38]. Alignment of the *TRI5* and the *TRI1/16* clusters for these three species revealed the presence of full length copies of the *TRI7*, *TRI13* and *TRI16* genes which confirmed *F. venenatum* to encode *TRI* clusters reminiscent of type A trichothecene biosynthesis. Hence, as previously found, the phylogenetic relationship of the *TRI* clusters does not correlate with the species phylogenetics.

A second inter-species comparative analysis identified the presence or absence of pre-identified *F. graminearum* *TRI6* targets previously determined by ChIP-Seq [39], in *F. venenatum* using BLASTP. The transcription factor *TRI6* is known to be a global regulator of gene expression in *F. graminearum* both *in planta* and when the fungus is growing under specific mycotoxin inducing conditions in vitro [39]. Fourteen of the *F. graminearum* *TRI6* targets were found to have a low alignment score of below the query and target threshold of 55% in *F. venenatum*. These included the following targets: a P450 gene described as involved in secondary metabolism (FGRRES_17130), the *TRI7* gene which is truncated in *F. graminearum* PH-1 but functional in *F. venenatum* (FGRRES_03533*)*, two genes linked to carbon metabolism (FGRRES_16885 and FGRRES_17740), one gene linked to nitrogen metabolism and energy production (FGRRES_17446), and four genes linked to transcription/translation (FGRRES_03794_M, FGRRES_05017, FGRRES_07187_M and FGRRES_07751_M) and one to cell signalling (FGRRES_03108) (see Additional file 17).

### Comparison of the predicted *F. venenatum* and *F. graminearum* secretomes

The proteinaceous fungal secretome is of fundamental importance to determine how fungi interact with their environment, whether that be as a saprophyte or a pathogen. The secretion of hydrolytic enzymes is central to the acquisition of alternative nutrient sources by fungi [40]. Additionally, within a pathology context, small secreted proteins that promote a pathogenic interaction are described as fungal effectors [7]. The *F. venenatum* and *F. graminearum* secretomes were predicted using an identical pipeline [13], whilst the candidate effector repertoire was predicted using EffectorP [41]. Within both *Fusaria*, the secretome and putative effectors encoding genes were found to localise to chromosomal regions of low nucleotide homology (Fig. 1)*.* A sequence homology analysis revealed that most secreted proteins and putative effectors to be conserved between the two *Fusaria* (see Additional file 18)*.* Therefore, despite the very different lifestyles, most of the secretome (using a cutoff of 50% coverage from a global alignment for both target and query) was present in both the pathogenic and saprophytic *Fusaria.* This suggests that only a few secreted proteins are specifically involved in the pathogenic lifestyle, while the majority are required for the completion of the disease cycle on plant biomass. *F. graminearum* had a lower number of species-specific (Fg vs Fv) secretome proteins (109 vs 151 < 50% coverage, 175 vs 247 < 70% coverage), with the number of secreted hydrolytic enzymes and species-specific secreted hydrolytic enzymes accounting for some of this increase in *F. venenatum* (Fig. 2, Additional file 19). The functional profiles of the species-specific secretomes were very distinct, with the *F. venenatum-*specific secretome predominantly presenting proteins involved in carbohydrate catabolic and metabolic processes, while the *F. graminearum-*specific secretome identified proteins involved in oxidation, reduction and pathogenesis-related processes (Fig. 3). The species-specific (Fg vs Fv) secreted proteins annotated to be potentially involved in pathogenesis were also defined as putative effectors, by EffectorP. These included an endo-beta-xylanase and all four KP4 killer toxins present in *F. graminearum* of which three have adjacent loci. *F. venenatum* does have three KP4 killer toxin genes annotated (FVRRES_4386, FVRRES_4580 and FVRRES_4581) but these are highly divergent to the *F. graminearum* set and only two remain clustered. The *F. graminearum* secreted proteins involved in oxidation-reduction (i.e. EC:1.1.1.158), not present in *F. venenatum* using a threshold of 50% coverage, included two flavin-adenine dinucleotide (FAD) binding proteins (FGRRES_15982 and FGRRES_10609), and a FAD-linked oxidase (FGRRES_10611). These species*-*specific differences could potentially reflect adaptations of the two *Fusaria* to saprophytic and pathogenic lifestyles.

### The minimal *F. graminearum* gene set specific to a pathogenic lifestyle

The identification of the minimal gene set specific to the pathogenic lifestyle in *Fusaria* represents a powerful tool and a novel dataset for the further evaluation of *F. graminearum*. An updated set of *F. graminearum* species-specific genes in relation to NCBI with the addition of *F. culmorum* UK99, *F. poae*, and *F. venenatum* revealed a reduced set of 690 genes from the original 741 reported by King et al. [25] using a cutoff of 50% identity from a global alignment for both target and query coverage (see Additional file 14). Of these 690 genes, five are predicted to be secreted in version 4.3 of the annotation analysed herein, a recent secretome update (annotation version 5.0 [42]) has increased this to 11 with eight as putative effectors, but again with no annotation, and 39 genes are found within predicted secondary metabolite clusters. A previous comparative genomic study which included three pathogenic *Fusarium* species, namely *F. graminearum*, *F. oxysporum* f. sp. *lycopersici* and *F. verticillioides*, identified 75 genes to be specific to pathogenic fungi and predominately absent from non-pathogenic fungi, while exhibiting signatures of diversifying selection pressure [43]. The majority of these gene lacked any functional annotation and none in *F. graminearum* were considered to code for transcription factors. The *F. venenatum* genome was used to further refine this classification. In total, 15 *F. graminearum* genes could no longer be classified as pathogenicity-associated, due to their presence in *F. venenatum* with greater than 50% target/query coverage, reducing the number to 60, while a further 44 *F. graminearum* pathogenicity-associated genes had less than 50% target/query coverage vs *F. venenatum*. Only 16 of the originally classified *F. graminearum* pathogenicity-associated genes did not have a BLASTP hit and so were not present in *F. venenatum* (Table 3, see Additional file 20) [43, 44].

**Table 3:** The 16 *F. graminearum* vs *F. venenatum* species-specific pathogenicity genes, reduced from the 75 found in a prior intergenomic comparison of *F. graminearum*, *F. oxysporum* f. sp. *lycopersici* and *F. verticillioides*

Version 4.0 ID	Version 5.0 ID	Blast description	InterPro description
FGRRES_00521	FGRAMPH1_01T01329	hypothetical protein FGSG_00521	N/A
FGRRES_02618	FGRAMPH1_01T06281	hypothetical protein FGSG_02618	N/A
FGRRES_02904	FGRAMPH1_01T11581	hypothetical protein FGSG_02904	N/A
FGRRES_03222	FGRAMPH1_01T12357	hypothetical protein FGSG_03222	N/A
FGRRES_04462	FGRAMPH1_01T15361	hypothetical protein FGSG_04462	N/A
FGRRES_04840	FGRAMPH1_01T16451	peptidase c14 caspase catalytic subunit p20	Caspase-like domain
FGRRES_05785	FGRAMPH1_01T18757	hypothetical protein FGSG_05785	N/A
FGRRES_06601_M	FGRAMPH1_01T22679	unnamed protein product	N/A
FGRRES_08267	FGRAMPH1_01T09515	hypothetical protein FGSG_08267	N/A
FGRRES_10593	FGRAMPH1_01T08485	hypothetical protein FGSG_10593	N/A
FGRRES_11016	FGRAMPH1_01T21023	hypothetical protein FGSG_11016	N/A
FGRRES_12623_M	FGRAMPH1_01T16415	LOW QUALITY PROTEIN: hypothetical protein FGSG_12623	N/A
FGRRES_12656	FGRAMPH1_01T16757	immunoglobulin variable region used by the itc63b heavy chain	N/A
FGRRES_13187	FGRAMPH1_01T25375	hypothetical protein FGSG_13187	N/A
FGRRES_13517	FGRAMPH1_01T27893	hypothetical protein FGSG_13517	IQ motif, EF-hand binding site
FGRRES_13534	FGRAMPH1_01T27583	hypothetical protein FGSG_13534	N/A

The multi-species pathogen-host interactions database (PHI-base) contains ~ 8600 curated interactions and ~ 4750 genes from peer reviewed literature, providing phenotypic data associated with genetic mutations in pathogens and their impact on virulence, such as loss of pathogenicity, reduced virulence and hypervirulence [45, 46]. The PHI-base homologues in the *F. venenatum* and *F. graminearum* genomes were identified and subdivided according to the impact caused by their absence, or reduced function, on the respective pathogen-host interaction (Table 4, Additional file 21). *F. graminearum* had a higher number of PHI-base homologues than *F. venenatum* due to the large number of *F. graminearum* entries within PHI-base (*n* = 976 genes). However, the overall number of PHI-base homologues defined as being experimentally proven to be involved in virulence, i.e. conferring either a loss of pathogenicity, reduced virulence, increased virulence or an effector phenotype, was comparable for both species. Only two major exceptions were identified, namely the loss of the transcription factors GzZC120 (FGRRES_08028_M) and GzZC305 (FGRRES_00147) (see Additional file 21) whose experimental deletion in *F. graminearum* resulted in reduced virulence on wheat heads, but no other altered phenotypes [47]. These two predicted transcription factors reside in regions of the *F. venenatum* genome with a high frequency of transposon sequences. None of the PHI-genes associated with ‘loss in pathogenicity’ annotations were missing from the *F. venenatum* genome. This demonstrates that the virulence profile of genes known to be involved in pathogenicity are very similar in the two *Fusaria*. However, the combined loss of these two genes, and potentially others (discussed later), may be important to the lack of pathogenicity*.*

**Table 4:** The conservation of PHI-base homologues in *F. venenatum* and *F. graminearum*

Phenotype^a^	*F. venenatum*		*F. graminearum*	
	70%^c^	50%	70%	50%
All	679	969	830	1064
Loss of pathogenicity	15	43	13	40
Reduced virulence	162	234	163	240
Effector	0	5	0	5
Increased virulence/enhanced antagonism	7	11	7	11
Lethal^b^	51	76	66	89

^a^Included is the phenotypic impact of the absence of the PHI-base gene in *F. graminearum*.

^b^Presumed to be an ‘essential for life’ gene because of the lack of transformants recovered in a reverse genetics experiment reported in the peer reviewed literature

^c^50%/70% target and query coverage from a BLASTP alignment

For *F. graminearum* a considerable number of genes clusters have either been predicted or demonstrated to be responsible for the production of a range of secondary metabolites [33]. Genes predicted to reside within fungal secondary metabolite gene clusters (SMC) include polyketide synthetases (PKS) and nonribosomal peptide synthetases (NPS) often in association with transcription factors. Additions to the SMC clusters within the *F. venenatum* and *F. graminearum* genomes were predicted using the AntiSmash [48] and SMURF software [49], using the prior predictions for *F. graminearum* from Sieber et al. [33] and *F. venenatum* BLASTP hits and positional information as a baseline. In total, the *F. venenatum* and *F. graminearum* genomes encoded 60 and 69 predicted secondary metabolite clusters (an additional 2 versus *Sieber* et al), respectively, of which six were specific to *F. venenatum* and fifteen were specific to *F. graminearum* (Fig. 1, Additional file 16)*.* Both *Fusaria* possessed the biosynthetic machinery to produce, secrete and subsequently import the non-toxic iron scavenging extracellular siderophore TAFC. The putative secondary metabolites specific to the pathogenic species, *F. graminearum*, included two transcription factor containing clusters (C1, C67), two HC-toxin annotated clusters (C2, C61), seven PKS clusters with annotations of prostacyclin, fusarielin, orcinol/orsellinic acid, daunorubicin and the zearalenone mycotoxin (C15, C16, C18, C31, C32, C47, C60) and four NPS containing clusters (C6, C62, C64, C66) (Fig. 1). Due to the changes in gene predictions for the *F. graminearum* genome (King et al., 2015, [25]) since the Sieber et al. analysis, seven of the *F. graminearum*-specific SMC could be expanded in gene content (C02, C16, C31, C32, C47, C60, and C64), whilst nine SMCs identified in both *F. graminearum* and *F. venenatum* were expanded in content (C13, C22, C27, C33, C34, C37, C42, C53, C63) (see Additional file 16)*.* The *F. venenatum*-specific secondary metabolite clusters included the beta-ketoacyl synthase (VC1), lovastatin (VC2), enniatin (VC5) and equisetin (VC6) clusters and a transcription factor and PKS containing clusters (Fig. 1). The species-specific secondary metabolite clusters in both *Fusaria* were found in regions of low homology proteins/nucleotides, suggesting their localisation within these genomic regions was important to the evolution of novel functions specific to a particular lifestyle. Finally the GC contents of the orthologous clusters were inspected and found to be very similar (median GC content of 49.43 and 49.5% for *F. graminearum* and *F. venenatum*, respectively) (see Additional file 16).

### Expression of the minimal pathogenesis-specific *F. graminearum* gene set during infection

**Fig. 5:**
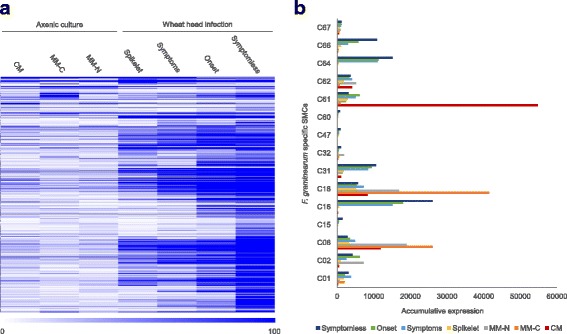
Analysis of the *F. graminearum* specific gene set. **a** The expression of the *F. graminearum* specific genes during the distinct phases of wheat head infection and axenic culture. Note that 855 *F. graminearum* specific genes were more highly expressed during infection than in vitro culture. **b** The accumulative expression of the *F. graminearum*-specific secondary metabolite gene clusters during the distinct phases of wheat head infection and axenic culture. Wheat infection phases included i) symptomless infection, ii) the onset of symptoms, iii) fully symptomatic tissue, and iv) the inoculated fully symptomatic spikelet from where infection originated [44]. In vitro cultures included complete media (CM), minimal media without carbon (MM-C), and minimal media without nitrogen (MM-N)

Over the past 10 years, transcriptome studies using the same Affymetrix array [50], have investigated various saprophytic, developmental and in planta phases of the *F. graminearum* lifecycle on various substrates and host species [51]. In two of the most recent studies the early symptomless and late symptomatic phases of the wheat infection process have been explored during juvenile wheat coleoptile and mature wheat head colonisation and have been used to define the spatial temporal coordination of virulence mechanisms during infection [44, 50, 52]. In total, 690 (395 identified on microarray) genes were found to be specific to *F. graminearum* when compared using the set predicted in King et al. [25] using NCBI, plus the *F. venenatum*, *F. poae*, and *F. culmorum* UK99 predicted proteomes*.* These 395 genes were found to be up-regulated during mature wheat head infection, with the majority (370 genes) showing increased transcript abundance during symptomless wheat head infection, implicating them as potentially being involved in the establishment of disease (Fig. 5, Additional file 22). Interestingly, five of the seven SMC identified to be highly (C16, C31, C64 and C66) or moderately (C02) expressed during the symptomless phase of wheat head colonisation by *F. graminearum* were found to be absent or highly divergent within the *F. venenatum* genome (Fig. 5, Additional file 22). Whilst the two other *F. graminearum* clusters C48 and C47 were predicted to be present in *F. venenatum.* The combination of comparative genomics between closely related pathogenic and non-pathogenic *Fusaria* has therefore identified novel *F. graminearum* genes that are not present in a non-pathogenic relative, and highly expressed during wheat infection, implicating them as potentially contributing to virulence.

## Discussion

The *Fusaria* represent a large group of economically destructive, mycotoxigenic and non-mycotoxigenic pathogens of plants and animals, in addition to soil dwelling saprophytes often used as microbial cell factories for the production of myco-protein or industrial heterologous proteins [1, 2, 16–22]. Comparative genomics is proving to be an essential tool in the identification of the fungal determinants of a pathogenic lifestyle and the occupancy of a particular niche [24, 53]. This study has confirmed that *F. venenatum* is non-pathogenic when tested on wheat plants and tomato fruit. Herein, we present the interspecies genomic comparison of *F. venenatum*, a soil-dwelling saprophyte, and the plant pathogen *F. graminearum*, the etiological agent of FHB disease on cereal crops. This bioinformatics study has revealed striking similarities in the physical organisation and functional content of the genomes of these two closely related *Fusaria.* This raises the questions, why is *F. venenatum* not a pathogen, or conversely, what makes *F. graminearum* a pathogen? One notable distinction between the two genomes was the increased quantity of repetitive elements and transposons in the *F. venenatum* genome, plus the absence of the *MAT1–2* required for homothallic sexual reproduction*.* This requirement for a complementary sexual partner could increase the retention of repeats. Overall, the genome organisation was very similar between the two *Fusaria*, with four large chromosomes and centromeres in similar genomic locations, although the predicted centromeres were 25% smaller in size in *F. venenatum*. The predicted proteome for *F. venenatum* was slightly smaller (13,946 vs 14,164), potentially due to some non-annotated genes in this first draft genome, and the GC content (47.6%) is in line with other *Fusarium* species. Overall, to date the *F. venenatum* genome is the most closely related genome, of a non-pathogenic species, to the important pathogen *F. graminearum.*

The gene synteny between the two species was very high, except for a 2010 Kbp region on chromosome 3 that contains 783 annotated genes in v4.0 and 786 in v5.0. Within this rearrangement, the C33 ferricrocin secondary metabolic cluster is located. However, this rearrangement did not lead to any gene disruptions. A similar chromosome rearrangement is found in the pathogenic species *Fusarium poae*, suggesting that this rearrangement predates the speciation of *F. poae* and *F. venenatum*. The other seven chromosomal rearrangements were small in size and were located solely in sub-telomeric regions, or in two cases, in regions predicted to be the sites of ancient chromosome fusion events [23].

The clear majority of the predicted proteins and their functions were conserved between *F. venenatum* and *F. graminearum*. In a pair-wise analysis 50% of the predicted proteome at < 70% coverage were considered to be species*-*specific or highly divergent. However, in a global multiple species analysis using 10^− 5^ and 10^− 20^ E-value cutoffs, 786 (99% with no BLASTP annotation) and 1149 (94% with no BLASTP annotation) of the *F. venenatum* genes were predicted to be unique to this species. The species-specific genes in both *Fusaria* are distributed throughout their genome. These results highlight the value of using a comparative study of mulitiple closely related *Fusarium* species to refine the identification of genes specific to a pathogenic or saprophytic lifestyle, with each addition increasing the specificity.

Both the *F. venenatum* A3/5 and *F. graminearum* PH-1 strains possess the capacity to produce trichothecene mycotoxins. However, during the fermentative conditions of Quorn production no detectable trichothecenes are present [15]. The genomic comparison revealed the trichothecene mycotoxin *TRI5* gene cluster and the *TRI1/TRI16* gene cluster to reside at the same chromosomal locations and in the same microsyntenic gene contexts in both species (Fig. 4). This consistent chromosome position and flanking gene microsynteny is also true for *TRI101, TRI15* and *TRI15R*. The presence of functional copies of *TRI7*, *TRI13* and *TRI16* in *F. venenatum* but inactive truncated copies of all three genes in *F. graminearum*, accounts for *F. venenatum* producing type A, and *F. graminearum* type B, trichothecenes.

In the *Fusaria,* the trichothecene types are divided according to the absence (type A) or presence (type B) of a keto group at the C-8 position on the trichothecene ring. Both type A trichothecenes, such as DAS and T-2 (produced by *F. venenatum* and *F. sporotrichoides*, respectively), and type B trichothecenes, such as DON and NIV (produced by *F. graminearum*), are described as being phytotoxic [38]. Trichothecenes are ribotoxic and induce translational arrest. The T-2 mycotoxin activates MAP kinase signalling and induces cell death in both animals and plants. However, differences in host-susceptibility to specific trichothecenes exist, as unlike the T-2 mycotoxin, DON does not induce cell death at concentrations sufficient to induce translational arrest [38]. In fact, in plant cells, low and high concentrations of DON respectively inhibit apoptosis or induce host cell death [54, 55]. During the establishment of symptomless *F. graminearum* infections of wheat the main *TRI* cluster is dramatically up-regulated, while the absence of the ability to produce DON results in an enhanced host response and reduced virulence [11, 12, 44]. Therefore, the toxigenic differences between DAS-producing *F. venenatum* and DON-producing *F. graminearum* potentially contribute to the opposing lifestyles of these closely related *Fusaria.*

Previous phylogenetic studies have found that differences in trichothecene production do not correlate with the general species phylogeny, while polymorphisms within the trichothecene biosynthetic genes are trans-specific, persist through multiple speciation events, and are maintained by balancing selection [56]. Here, the *TRI* cluster of *F. venenatum* was reminiscent of other type A trichothecene producers, such as *F. sporotrichioides* and *F. langsethiae*, while the rest of the *F. venenatum* genome showed a far greater reciprocal homology to DON producer *F. graminearum.* The trichothecene clusters either evolved by vertical inheritance or were transferred horizontally within *Fusaria* via the transfer of chromosomes between species, or interspecific hybridisation. Horizontal gene transfer presents a mechanism by which fungal genomes can acquire new gene clusters, as may be the case for several other secondary metabolite clusters in *F. graminearum* [33, 53]. However, there is no evidence of this among *TRI* genes due to the lack of co-linearity across the non-mycotoxin biosynthetic genes flanking the *TRI* cluster in trichothecene and non-trichothecene producers, such as *F. graminearum* and *F. oxysporum* [38]. In contrast, *TRI101* resides outside the *TRI* cluster and is flanked by the *PHO5* and *URA7* genes in trichothecene producing *Fusaria*, while the microsynteny of this region is conserved in non-trichothecene producers, such as *F. oxysporum* and *F. fujikuroi*, where *TRI101* is dysfunctional [38]. This suggests that *TRI101*, which is involved in trichothecene biosynthesis and in a self-protection mechanism, was inactivated by the accumulation of mutations, which impacted upon the capacity that produces and withstands trichothecene production. This mechanism of evolution appears to contribute to the distinct trichothecene types in *F. venenatum* and *F. graminearum*, as the microsynteny across the region is high, yet *TRI7*, *TRI13* and *TRI16* have been inactivated, diverting secondary metabolism towards the biosynthesis of type B trichothecenes in *F. graminearum*.

The pathogenic profile of a species, as determined by the identification of PHI-base homologues, provides an insight into a species virulence repertoire. This revealed both *F. venenatum* and *F. graminearum* possess a virtually identical set of PHI-base genes, with *F. venenatum* missing two loss-of-virulence but no loss-of-pathogenicity annotated genes. Interestingly the two missing/highly divergent PHI-base genes are predicted to be transcription factors which when deleted in *F. graminearum* lead to reduce virulence towards wheat heads, but no other altered phenotype [47]. Species-specific genes beyond the known virulence factors, such as the *TRI* and PHI-base genes, identified a minimal set of pathogenesis-specific genes of unknown function in *F. graminearum*, where 855 genes were induced during wheat infection, of which 586 were up-regulated during symptomless infection and the establishment of disease. Similarly, five *F. graminearum-*specific secondary metabolite gene clusters, which were absent from the *F. venenatum* genome, were co-ordinately induced during wheat infection.

## Conclusions

This comparative genomics study of a closely related non-pathogen and pathogen has provided valuable information on the evolutionary relationship between these two *Fusaria* and insights into adaptations for a pathogenic lifestyle. This refinement of *F. graminearum-*specific genes associated solely with a pathogenic lifestyle will facilitate the identification of novel genes and gene clusters involved in pathogenesis and the development of novel FHB disease control strategies.

## Methods

### Origin and maintenance of Fusarium strains

The American type culture collection number of *F. venenatum* A3/5 is ATCC 20334™. The strain was kindly provided by G. D. Robson (British Mycological Society, Manchester, UK). *F. graminearum* PH-1 (FGSC 9075, origin USA) and *F. culmorum* FcUK99 (FGSC 10436, origin UK) are available from the Fungal Genetics Stock Center, Kansas City, MO, USA [56]. *F. graminearum* Fg602 is a UK field strain isolated from diseased wheat in 2006 at Rothamsted Research. All *Fusarium* strains including *F. venenatum* A3/5 were routinely propagated on carrot agar plates [36]. Carrot agar was found to support high asexual spore production for *F. venenatum* throughout this study*.*

### Biological tests

Plant infection and pathogenicity tests on tomato fruits cultivar Moneymaker and wheat (*Triticum aestivum*) plants of the fully susceptible cultivar Bobwhite followed previously established protocols [57]. For the co-inoculation of *F. venenatum* A3/5 with the FHB pathogen *F. culmorum* isolate FcUK99 the wheat inoculation protocol was modified as follows. For spore droplet co-inoculation, equal amounts of spores at 5 × 10^4^ spores/ml in water were mixed. Five ul of the spore mix was pipetted into two spikelets in the mid-section of the wheat spike at Zadoks growth stage 63 [58]. Disease progression was monitored over 20 days. For the *F. venenatum* pre-spraying experiments, wheat spikes were sprayed, when half the wheat spike emerged from the surrounding flag leaf (Zadoks growth stage 48), approximately three days before inoculation with the FHB pathogen *F. culmorum*.

### *Fusarium venenatum* A3/5 genomic DNA preparation

Genomic fungal DNA for sequencing was extracted using the CTAB protocol [59] and purified using a Qiagen Kit (Qiagen Ltd., Crawley, West Sussex, UK). High-quality genomic DNA was then submitted to the Earlham Institute (Norwich, UK) for generation of a 0.8 kb fragment library. The Illumina HiSeq 2000 sequencing platform (San Diego, CA) was used to produce 100 bp paired-end reads [60].

### Assembly and alignment

Reads were not pre-processed. For de novo assembly, the software SOAPdenovo2 (version 2.0.4) was used with different k-mer values: 61–91. Reference sequence statistics were extracted from Tablet and Geneious [61] (version 9.1 created by Biomatters). Lastz (version 1.02.00) and Mauve (version 2.3.1) was used from within Geneious to align genomic sequences to *F. graminearum* (ENA study accession: PRJEB5475).

### Genome annotation

The assembled genome was annotated using the MAKER (version 2.30) [30] annotation pipeline with RepeatMasker (version 4.0.5) [62]. A *F. venenatum* specific repeat library was constructed using RepeatModeler (version 1.0.7) and supplied to MAKER for the repeat masking step. Gene calls were generated using FGENESH (version 3.1.2) [63] using the *Fusarium* matrix, AUGUSTUS (version 2.7) [64] using *F. graminearum* species model, GeneMark [65] and SNAP [66], with evidence taking *Fusarium* reviewed proteins in UNIPROT with the keyword “*Fusarium*”, ESTs [67], and trinity assemblies using RNA-seq from the mycelium and spores of PH-1, and wild type Z-3639. This evidence was provided to MAKER as hints to the annotation. Non-coding RNA were identified using default settings with both tRNAscan-SE-1.3.1 [68] and Infernal-1.1 [69].

### Gene statistics, Interproscan domain, GO and enzyme comparisons

Blast2GO V.3.1 was used with Decypher BLASTP searches with an E-value of 0.001 against the NCBI nr database from 27/06/15, filtered using blast2go annotation algorithm with settings, E-value filter 0.000001, Annotation CutOff 55, GO weight 5, Hsp-Hit Coverage CutOff 0, and GO and enzyme code annotated using a local GO database from 07/2015. Interproscan results were imported into blast2go and the GO annotations merged. Annotation statistics were produced using Eval V.2.2.8 and Geneious. The “Assign your proteins to OrthoMCL Groups” tool was used with the predicted protein sequences from the OrthoMCL website [70] to assign paralogue groups. BUSCO V1.1b1 [71] was used with the 1438 core fungal genes with both *Fusaria* genomes using default settings.

### Secretome identification and effector prediction

Interproscan-55.0 was used to identify signal and transmembrane domains. Proteins smaller than 20 amino acids were excluded. ProtComp (Version 9.0) (Softberry, USA) and result columns, LocDB and PotLocDB were used to exclude GPI anchored membrane proteins and other non-extracellular loci proteins. WoLfPSort [72] (extracellular score > 17, WoLfPSort) was used to identify final destination and big-PI [73] to further remove GPI-anchored proteins. Putative effector proteins were predicted using EffectorP (v1.0).

### Secondary metabolite cluster analyses

SMURF [49] and antiSMASH [48] were used to predict and complement prior published secondary metabolite clusters [33]. Percentage GC content for each cluster were calculated by extracting each clusters gene sequence, including introns, using bedtools, and GC content calculated using EMBOSS infoseq [74]. Median percentage GC content values were taken using Excel.

### Phylogenetic tree

The BUSCO V1.1b1 fungi database was used with the proteomes of *F. solani, F. oxysporum, F. verticillioides, F. mangiferae, F. fujikuroi, F. proliferatum, F. poae, F. venenatum, F. langsethiae, F. graminearum* and *F. pseudograminearum* taken from Ensembl Fungi release 38. The resulting “complete” proteins across the species, subtracting for those that had more than one hit with a BUSCO gene were 904. For each species the proteins were concatenated and an alignment done using MAFFT (V7.222) [75] in Geneious. RAxML blackbox [76] was used to create a phylogenetic tree with 100 bootstraps, JTT substitution matrix, a Gamma model of rate heterogeneity, and using a maximum likelihood search. The substitution rate is on each branch and 100% branch support was found from 100 bootstraps.

### Data access

Sequence Read Archive (SRA) sample accession numbers for Illumina sequences is ERS568866. The RRes genomic and mitochondrial sequence for *F. venenatum* and *F. graminearum* are available at the European Nucleotide Archive (ENA Submission) with respective study accessions: PRJEB7533 and PRJEB5475.

## Additional files

Additional file 1: Biological growth characteristics of *F. venenatum* compared to its close pathogenic relative *F. graminearum*. (A) Appearance of inoculated wheat head of cv. Bobwhite, 21 days after plug inoculations (dpi) with *F. venenatum* (Fv), (B) mock inoculation with water, (C) with *F. graminearum* (Fg). The white arrow indicates the inoculation points. (D) Appearance of wheat head 16 days after spray inoculation (dsi) with Fv, (E) mock spray inoculation with water, (F) spray inoculation with Fg. (G) Wheat seeds collected from a typical spray inoculated wheat head at 21 dsi with Fv, (H) mock spray with water, (I) with Fg. (J) Fv macrospores, (K) Fg macrospores. (L) Fv and (M) Fg growth on PDB plate at 6 days. (N) Fv inoculated tomato fruit at 4 days, (O) water control, (P) Fg. (Q) Fv inoculated tomato fruit at 12 days, (R) water control, (S) Fg. Bars in J, K: 10 μm. (PDF 268 kb)

Additional file 2: Co-inoculation of *F. venenatum* with plant-pathogenic Fusaria in vitro and in vivo*.* (A) Carrot agar plate inoculated with agar plugs of strains Fv A3/5, two isolates of Fg (PH1 and 602), and Fc UK99. A barrage line between strains is visible at day 6. (B) *F. culmorum* and *F. venenatum* spores where mixed in equal amounts and co-inoculated into two spikelets at anthesis. As a control, water was inoculated (C) *F. venenatum* spores were sprayed onto wheat heads at the booting stage to potentially prime plant defence responses, followed by point-inoculation with *F. culmorum* spores of two heads per spike" should be "two spikelets per head at anthesis. (D) Wheat plants were treated as in C. Just after inoculaton with *F. culmorum*, wheat heads were sprayed once more with *F. venenatum* spores until run-off. Error bars show standard deviation, *n* = 6. (PDF 111 kb)

Additional file 3: BUSCO analysis of proteomes for selected *Fusaria*. (XLS 1371 kb)

Additional file 4: A phylogenetic tree of 904 identified common proteins (see Additional file 3) using *F. solani, F. oxysporum, F. verticillioides, F. mangiferae, F. fujikuroi, F. proliferatum, F. poae, F. venenatum, F. langsethiae, F. graminearum* and *F. pseudograminearum*. The substitution rate is on each branch and 100% branch support was found from 100 bootstraps. Type A, B, or A/B trichothecene producers were labelled. The tree was rooted to *F. solani*. (PDF 104 kb)

Additional file 5: Comparative genomic analysis of the *Fusarium venenatum* genome with other fungi. This reveals close similarity to the pathogenic species *F. graminearum* and *F. pseudograminearum*. (XLS 37 kb)

Additional file 6: Positions and lengths of *Fusarium venenatum* and *Fusarium graminearum* centromeres. This shows a 25% decrease in average size of the *F. venenatum* centromeres compared to *Fusarium graminearum*. (XLS 43 kb)

Additional file 7: Comparison of the reference *F. venenatum* and *F. graminearum* genome sequences per individual chromosome (Chr) or supercontig (FV_1_1). Detailed comparisons of bp length and ‘N’ base content. (XLS 36 kb)

Additional file 8: Comparative statistics on the number of coding and non-coding gene models predicted in the *Fusarium venenatum* (Fv) and *F. graminearum* (Fg) genomes. (XLS 29 kb)

Additional file 9: *Fusarium graminearum* alignment and gene ID’s found in the inverted/translocated regions A/B/C in relation to *Fusarium venenatum*. Mauve alignments in Sheet 1 of *Fusarium venenatum* and *F. graminearum* chromosome 3. The small blue inversion/translocation in *Fusarium graminearum* has 56 genes, the small green translocation 144 genes, and the large blue/pink inversion/translocation has 586 genes (see Sheet 2–4). Below the top mauve alignment are *F. venenatum* and *F. poae* chromosome 3 aligned to *F. graminearum* using Lastz. (XLS 678 kb)

Additional file 10: Classes of transposon and repeat found in *Fusarium graminearum* and *Fusarium venenatum*. (XLS 37 kb)

Additional file 11: The mating locus within the *F. venenatum* and *F. graminearum* genomes. *F. venenatum* A3/5 contains only the *MAT1–1* locus confirming heterothallism and the requirement for a complementary sexual partner. Whilst homothallic *F. graminearum* possesses both the *MAT1–1* and *MAT1–2* loci and hence does not require a complementary partner for sexual reproduction. Gene designations: A) FGRRES_08887, B) FGRRES_08888_M, C) FGRRES_08889_M, D) FGRRES_08894, E) FVRRES_05564, F) FGRRES_15525, G) FGRRES_13273_M. (PDF 90 kb)

Additional file 12: *Fusarium venenatum* species-specific genes. An analysis of genes that lack a BLASTP top hit from NCBI nr database using 10^− 6^ and 10^− 20^ E-value cutoffs. (XLS 3165 kb)

Additional file 13: A figure locating the positions of *Fusarium venenatum* species-specific genes versus NCBI, in relation to sequences predicted to be the secretome, tRNA, and transposons. (PDF 144 kb)

Additional file 14: Species-specific proteins between *F. venenatum* (Fv) and *F. graminearum* (Fg), identified using BLASTP analysis of both Fv and Fg proteomes. (XLS 4938 kb)

Additional file 15: Paralogue protein groups in *Fusarium venenatum*. Some groups show adjacent (neighbour) groupings representing recent duplications, whilst others represent near neighbours (< 40 genes) and others more distant duplications (> 100 genes). (XLS 38 kb)

Additional file 16: A summary of secondary metabolite clusters (SMCs) found in *Fusarium venenatum* and *F. graminearum*. Using Sieber et al. [33] 67 predictions as a template, SMURF and ANTISMASH were used to refine SMC predictions to 69 Fg SMCs and an additional 6 for Fv, combined with BLASTP of respective proteomes to identify presence in each species. (XLS 2940 kb)

Additional file 17: *Fusarium venenatum* presence of TRI6 *Fusarium greaminearum* binding sites predicted by Nasmith et al. [39]. *Fusarium venenatum* BLASTP alignment percentages were added to identify presence or absence. (XLS 61 kb)

Additional file 18: *Fusarium graminearum* and *F. venenatum* secretome predictions and respective presence. (XLS 479 kb)

Additional file 19: Annotation of *Fusarium venenatum* and *Fusarium graminearum* secretomes (A) reveals striking similar functional profiles. The enzymatic repertoire of *F. venenatum* and *F. graminearum* including secreted enzymes (B) and species-specific secreted enzymes (C). (TIFF 83 kb)

Additional file 20: BLASTP analysis of previously identified pathogenicity factors. (XLS 37 kb)

Additional file 21: BLASTP analysis of both *Fusarium venenatum* and *F. graminearum* proteomes against PHI-base v4.3. (XLS 2317 kb)

Additional file 22: *Fusarium graminearum* specific genes and clusters and their expression during in vitro culture or during wheat head infection. (XLS 185 kb)

## Abbreviations

DAS: Diacetoxyscirpenol
DON: Deoxynivalenol
FHB: *Fusarium* head blight
HGT: Horizontal gene transfer
NCBI: National Center for Biotechnology Information
NPS: Nonribosomal peptide synthetase
PHI-base: Pathogen-Host Interactions database
PKS: Polyketide synthase clusters
RIP: Repeat induced point mutation
SMC: Secondary metabolite gene clusters
TAFC: Triacetylfusarinine C

## References

[1] Goswami RS, Kistler HC (2004). Heading for disaster: *Fusarium graminearum* on cereal crops. Mol Plant Pathol.

[2] Dean R, Van Kan JAL, Pretorius ZA, Hammond-Kosack KE, Di Pietro A, Spanu PD, Rudd JJ, Dickman M, Kahmann R, Ellis J (2012). The top 10 fungal pathogens in molecular plant pathology. Mol Plant Pathol.

[3] Roy KW, Rupe JC, Hershman DE, Abney TS (1997). Sudden death syndrome of soybean. Plant Dis.

[4] Di Pietro A, Madrid MP, Caracuel Z, Delgado-Jarana J, Roncero MIG (2003). *Fusarium oxysporum*: exploring the molecular arsenal of a vascular wilt fungus. Mol Plant Pathol.

[5] Nucci M, Anaissie E. *Fusarium* infections in immunocompromised patients. Clin Microbiol Rev. 2007;20(4):695–704. 10.1128/CMR.00014-07 PMC2176050 17934079

[6] Summerell BA, Leslie JF, Liew ECY, Laurence MH, Bullock S, Petrovic T, Bentley AR, Howard CG, Peterson SA, Walsh JL, Burgess LW. *Fusarium* species associated with plants in Australia. Fungal Divers. 2011;46:1–27.

[7] Brown NA, Hammond-Kosack KE, Gupta VK, Mach RL, Sreenivasaprasad S (2015). Secreted biomolecules in fungal plant pathogenesis. Fungal biomolecules: sources, applications and recent developments.

[8] Cuzick A, Urban M, Hammond-Kosack K (2008). *Fusarium graminearum* gene deletion mutants *map1* and *tri5* reveal similarities and differences in the pathogenicity requirements to cause disease on *Arabidopsis* and wheat floral tissue. New Phytol.

[9] Oide S, Moeder W, Krasnoff S, Gibson D, Haas H, Yoshioka K, Turgeon BG (2006). NPS6, encoding a nonribosomal peptide synthetase involved in siderophore-mediated iron metabolism, is a conserved virulence determinant of plant pathogenic ascomycetes. Plant Cell.

[10] Bluemke A, Falter C, Herrfurth C, Sode B, Bode R, Schaefer W, Feussner I, Voigt CA (2014). Secreted fungal effector lipase releases free fatty acids to inhibit innate immunity-related callose formation during wheat head infection. Plant Physiol.

[11] Brown NA, Urban M, Van De Meene AML, Hammond-Kosack KE (2010). The infection biology of *Fusarium graminearum*: defining the pathways of spikelet to spikelet colonisation in wheat ears. Fungal Biol.

[12] Brown NA, Bass C, Baldwin TK, Chen H, Massot F, Carion PWC, Urban M, van de Meene AML, Hammond-Kosack KE. Characterisation of the *Fusarium graminearum*-wheat floral interaction. J Pathogens. 2011;626345. 10.4061/2011/626345 PMC3335584 22567335

[13] Brown NA, Antoniw J, Hammond-Kosack KE (2012). The predicted secretome of the plant pathogenic fungus *Fusarium graminearum*: a refined comparative analysis. PLoS One.

[14] Brewer HC, Hammond-Kosack KE. Host to a stranger: *Arabidopsis* and *Fusarium* ear blight. Trends Plant Sci. 2015;20(10):651–63. 10.1016/j.tplants.2015.06.011 26440434

[15] O'Donnell K, Cigelnik E, Casper HH (1998). Molecular phylogenetic, morphological, and mycotoxin data support reidentification of the Quorn mycoprotein fungus as *Fusarium venenatum*. Fungal Genet Biol.

[16] Trinci APJ (1992). Myco-protein: a twenty-year overnight success story. Mycol Res.

[17] Trinci APJ (1994). Evolution of the Quorn® myco-protein fungus, *Fusarium graminearum* A3/5. Microbiology.

[18] Trinci APJ, Robson GD, Wiebe MG (2001). Evolution of *Fusarium venenatum* A3/5 in continuous flow (chemostat) culture. Fusarium: Paul E. Nelson Memorial Symposium.

[19] Royer JC, Moyer DL, Reiwitch SG, Madden MS, Jensen EB, Brown SH, Yonker CC, Johnstone JA, Golightly EJ, Yoder WT, et al. *Fusarium graminearum A* a 3/5 as a novel host for heterologous protein production. Biotechnology (N Y). 1995;13(13):1479–83. 10.1038/nbt1295-1479 9636307

[20] Berka RM, Nelson BA, Zaretsky EJ, Yoder WT, Rey MW, Arora DK, Khachatourians GG (2003). Genomics of *Fusarium venenatum*: an alternative fungal host for making enzymes. Applied Mycology & Biotechnology, fungal genomics.

[21] Berka RM, Rey MW, Brown KM, Byun T, Klotz AV (1998). Molecular characterization and expression of a phytase gene from the thermophilic fungus *Thermomyces lanuginosu*s. Appl Environ Microbiol.

[22] Wiebe MG, Robson GD, Shuster JR, Trinci APJ. pH regulation of recombinant glucoamylase production in *Fusarium venenatum* JeRS 325, a transformant with a *Fusarium oxysporum* alkaline (trypsin-like) protease promoter. Biotechnology and Bioengineering. 1999;64(3):368–372. 10.1002/(sici)1097-0290(19990805)64:3<368::aid-bit13>3.0.co;2-k 10397874

[23] Cuomo CA, Gueldener U, Xu J-R, Trail F, Turgeon BG, Di Pietro A, Walton JD, Ma L-J, Baker SE, Rep M (2007). The *Fusarium graminearum* genome reveals a link between localized polymorphism and pathogen specialization. Science.

[24] Ma L-J, van der Does HC, Borkovich KA, Coleman JJ, Daboussi M-J, Di Pietro A, Dufresne M, Freitag M, Grabherr M, Henrissat B, et al. Comparative genomics reveals mobile pathogenicity chromosomes in *Fusarium*. Nature. 2010;464(7287):367–73. 10.1038/nature08850 PMC3048781 20237561

[25] King R, Urban M, Hammond-Kosack MCU, Hassani-Pak K, Hammond-Kosack KE. The completed genome sequence of the pathogenic ascomycete fungus *Fusarium graminearum*. BMC Genomics. 2015; 10.1186/s12864-015-1756-1. 10.1186/s12864-015-1756-1 PMC4511438 26198851

[26] Vanheule A, Audenaert K, Warris S, van de Geest H, Schijlen E, Hofte M, De Saeger S, Haesaert G, Waalwijk C, van der Lee T. Living apart together: crosstalk between the core and supernumerary genomes in a fungal plant pathogen. BMC Genomics. 2016; 10.1186/s12864-016-2941-6. 10.1186/s12864-016-2941-6 PMC4994206 27552804

[27] Urban M, Cuzick A, Rutherford K, Irvine A, Pedro H, Pant R, Sadanadan V, Khamari L, Billal S, Mohanty S (2017). PHI-base: a new interface and further additions for the multi-species pathogen-host interactions database. Nucleic Acids Res.

[28] Pathogen Host Interactions (PHI-base). Rothamsted Research, Harpenden. 2017. http://www.phi-base.org. Accessed 21 Sept 2017.

[29] McCallum BD, Tekauz A, Gilbert J (2004). Barrage zone formation between vegetatively incompatible *Fusarium graminearum (Gibberella zeae)* isolates. Phytopathology.

[30] Holt C, Yandell M. MAKER2: an annotation pipeline and genome-database management tool for second-generation genome projects. BMC Bioinf. 2011; 10.1186/1471-2105-12-491. 10.1186/1471-2105-12-491 PMC3280279 22192575

[31] O'Donnell K, Rooney AP, Proctor RH, Brown DW, McCormick SP, Ward TJ, Frandsen RJN, Lysoe E, Rehner SA, Aoki T, et al. Phylogenetic analyses of RPB1 and RPB2 support a middle cretaceous origin for a clade comprising all agriculturally and medically important *Fusaria*. Fungal Genet Biol. 2013;52:20–31. 10.1016/j.fgb.2012.12.004 23357352

[32] Ward TJ, Bielawski JP, Kistler HC, Sullivan E, O'Donnell K. Ancestral polymorphism and adaptive evolution in the trichothecene mycotoxin gene cluster of phytopathogenic *Fusarium*. Proc Natl Acad Sci U S A. 2002;99(14):9278–83. 10.1073/pnas.142307199 PMC123131 12080147

[33] Sieber CMK, Lee W, Wong P, Muensterkoetter M, Mewes H-W, Schmeitzl C, Varga E, Berthiller F, Adam G, Gueldener U. The *Fusarium graminearum* genome reveals more secondary metabolite gene clusters and hints of horizontal gene transfer. PLoS One. 2014; 10.1371/journal.pone.0110311. 10.1371/journal.pone.0110311 PMC4198257 25333987

[34] Freitag M, Williams RL, Kothe GO, Selker EU. A cytosine methyltransferase homologue is essential for repeat-induced point mutation in *Neurospora crassa*. Proc Natl Acad Sci U S A. 2002;99(13):8802–7. 10.1073/pnas.132212899 PMC124379 12072568

[35] Galagan JE, Calvo SE, Borkovich KA, Selker EU, Read ND, Jaffe D, FitzHugh W, Ma LJ, Smirnov S, Purcell S (2003). The genome sequence of the filamentous fungus *Neurospora crassa*. Nature.

[36] Leslie JF, Summerell BA. The *Fusarium* laboratory manual. 1st ed. Ames: Blackwell Publishing Ltd; 2006.

[37] Zhang Y, Zhang K, Fang A, Han Y, Yang J, Xue M, Bao J, Hu D, Zhou B, Sun X, et al. Specific adaptation of *Ustilaginoidea virens* in occupying host florets revealed by comparative and functional genomics. Nat Commun. 2014; 10.1038/ncomms4849. 10.1038/ncomms4849 24846013

[38] Kimura M, Tokai T, Takahashi-Ando N, Ohsato S, Fujimura M. Molecular and genetic studies of *Fusarium* trichothecene biosynthesis: pathways, genes, and evolution. Biosci Biotechnol Biochem. 2007;71(9):2105–23. 10.1271/bbb.70183 17827683

[39] Nasmith CG, Walkowiak S, Wang L, Leung WW, Gong Y, Johnston A, Harris LJ, Guttman DS, Subramaniam R. Tri6 is a global transcription regulator in the phytopathogen *Fusarium graminearum*. PLoS Pathog. 2011; 10.1371/journal.ppat.1002266. 10.1371/journal.ppat.1002266 PMC3182926 21980289

[40] Brown NA, Ries LNA, Goldman GH. How nutritional status signalling coordinates metabolism and lignocellulolytic enzyme secretion. Fungal Genetics and Biology. 2014;72:48-63. 10.1016/j.fgb.2014.06.012 25011009

[41] Sperschneider J, Gardiner DM, Dodds PN, Tini F, Covarelli L, Singh KB, Manners JM, Taylor JM (2016). EffectorP: predicting fungal effector proteins from secretomes using machine learning. New Phytol.

[42] King R, Urban M, Hammond-Kosack KE. Annotation of *Fusarium graminearum* (PH-1) version 5.0. Genome Announc. 2017; 10.1128/genomeA.01479-16. 10.1128/genomeA.01479-16 PMC5256205 28082505

[43] Sperschneider J, Gardiner DM, Thatcher LF, Lyons R, Singh KB, Manners JM, Taylor JM. Genome-wide analysis in three *Fusarium* pathogens identifies rapidly evolving chromosomes and genes associated with pathogenicity. Genome Biol Evol. 2015;7(6):1613–27. 10.1093/gbe/evv092 PMC4494044 25994930

[44] Brown NA, Evans J, Mead A, Hammond-Kosack KE (2017). A spatial temporal analysis of the *Fusarium graminearum* transcriptome during symptomless and symptomatic wheat infection. Mol Plant Pathol.

[45] Urban M, Pant R, Raghunath A, Irvine AG, Pedro H, Hammond-Kosack KE (2015). The pathogen-host interactions database (PHI-base): additions and future developments. Nucleic Acids Res.

[46] Brown NA, Urban M, Hammond-Kosack KE (2016). The trans-kingdom identification of negative regulators of pathogen hypervirulence. FEMS Microbiol Rev.

[47] Son H, Seo YS, Min K, Park AR, Lee J, Jin JM, Lin Y, Cao PJ, Hong SY, Kim EK, et al. A phenome-based functional analysis of transcription factors in the cereal head blight fungus, *Fusarium graminearum*. PLoS Pathog. 2011; 10.1371/journal.ppat.1002310. 10.1371/journal.ppat.1002310 PMC3197617 22028654

[48] Weber T, Blin K, Duddela S, Krug D, Kim HU, Bruccoleri R, Lee SY, Fischbach MA, Mueller R, Wohlleben W (2015). antiSMASH 3.0-a comprehensive resource for the genome mining of biosynthetic gene clusters. Nucleic Acids Res.

[49] Khaldi N, Seifuddin FT, Turner G, Haft D, Nierman WC, Wolfe KH, Fedorova ND (2010). SMURF: genomic mapping of fungal secondary metabolite clusters. Fungal Genet Biol.

[50] Gueldener U, Seong K-Y, Boddu J, Cho S, Trail F, Xu J-R, Adam G, Mewes H-W, Muehlbauer GJ, Kistler HC. Development of a *Fusarium graminearum* Affymetrix GeneChip for profiling fungal gene expression *in vitro* and *in planta*. Fungal Genet Biol. 2006;43(5):316–25. 10.1016/j.fgb.2006.01.005 16531083

[51] Dash S, Van Hemert J, Hong L, Wise RP, Dickerson JA (2012). PLEXdb: gene expression resources for plants and plant pathogens. Nucleic Acids Res.

[52] Zhang XW, Jia LJ, Zhang Y, Jiang G, Li X, Zhang D, Tang WH. *In planta* stage-specific fungal gene profiling elucidates the molecular strategies of *Fusarium graminearum* growing inside wheat coleoptiles. Plant Cell. 2012;24(12):5159–76. 10.1105/tpc.112.105957 PMC3556981 23266949

[53] Gardiner DM, McDonald MC, Covarelli L, Solomon PS, Rusu AG, Marshall M, Kazan K, Chakraborty S, McDonald BA, Manners JM (2012). Comparative pathogenomics reveals horizontally acquired novel virulence genes in fungi infecting cereal hosts. PLoS Pathog.

[54] Diamond M, Reape TJ, Rocha O, Doyle SM, Kacprzyk J, Doohan FM, McCabe PF. The *Fusarium* mycotoxin deoxynivalenol can inhibit plant apoptosis-like programmed cell death. PLoS One. 2013; 10.1371/journal.pone.0069542. 10.1371/journal.pone.0069542 PMC3724914 23922734

[55] Desmond OJ, Manners JM, Stephens AE, MaClean DJ, Schenk PM, Gardiner DM, Munn AL, Kazan K. The *Fusarium* mycotoxin deoxynivalenol elicits hydrogen peroxide production, programmed cell death and defence responses in wheat. Mol Plant Pathol. 2008;9(4):435–45. 10.1111/j.1364-3703.2008.00475.x PMC6640518 18705859

[56] McCluskey K, Wiest A, Plamann M. The Fungal Genetics Stock Center: a repository for 50 years of fungal genetics research. J Biosci. 2010;35(1):119-26. 10.1007/s12038-010-0014-6 20413916

[57] Urban M, Mott E, Farley T, Hammond-Kosack K (2003). The *Fusarium graminearum* MAP1 gene is essential for pathogenicity and development of perithecia. Mol Plant Pathol.

[58] Zadoks JC, Chang TT, Konzak CF (1974). A decimal code for the growth stages of cereals. Weed Res.

[59] Doyle J, Doyle J (1987). A rapid DNA isolation procedure for small quantities of fresh leaf tissue. Phytochem Bull.

[60] Bentley DR, Balasubramanian S, Swerdlow HP, Smith GP, Milton J, Brown CG, Hall KP, Evers DJ, Barnes CL, Bignell HR (2008). Accurate whole human genome sequencing using reversible terminator chemistry. Nature.

[61] Kearse M, Moir R, Wilson A, Stones-Havas S, Cheung M, Sturrock S, Buxton S, Cooper A, Markowitz S, Duran C (2012). Geneious basic: an integrated and extendable desktop software platform for the organization and analysis of sequence data. Bioinformatics.

[62] Tempel S (2012). Using and understanding RepeatMasker. Methods Mol Biol.

[63] Solovyev V, Kosarev P, Seledsov I, Vorobyev D (2006). Automatic annotation of eukaryotic genes, pseudogenes and promoters. Genome Biol.

[64] Stanke M, Schoffmann O, Morgenstern B, Waack S. Gene prediction in eukaryotes with a generalized hidden Markov model that uses hints from external sources. BMC Bioinf. 2006; 10.1186/1471-2105-7-62. 10.1186/1471-2105-7-62 PMC1409804 16469098

[65] Lukashin AV, Borodovsky M (1998). GeneMark.hmm: new solutions for gene finding. Nucleic Acids Res.

[66] Korf I (2004). Gene finding in novel genomes. BMC Bioinf.

[67] Soanes DM, Skinner W, Keon J, Hargreaves J, Talbot NJ (2002). Genomics of phytopathogenic fungi and the development of bioinformatic resources. Mol Plant Microbe Interact.

[68] Lowe TM, Eddy SR (1997). tRNAscan-SE. A program for improved detection of transfer RNA genes in genomic sequence. Nucleic Acids Res.

[69] Nawrocki EP, Eddy SR (2013). Infernal 1.1: 100-fold faster RNA homology searches. Bioinformatics.

[70] Li L, Stoeckert CJ, Roos DS (2003). OrthoMCL: identification of ortholog groups for eukaryotic genomes. Genome Res.

[71] Simao FA, Waterhouse RM, Ioannidis P, Kriventseva EV, Zdobnov EM (2015). BUSCO: assessing genome assembly and annotation completeness with single-copy orthologs. Bioinformatics.

[72] Horton P, Park KJ, Obayashi T, Fujita N, Harada H, Adams-Collier CJ, Nakai K (2007). WoLF PSORT: protein localization predictor. Nucleic Acids Res.

[73] Eisenhaber B, Wildpaner M, Schultz CJ, Borner GH, Dupree P, Eisenhaber F (2003). Glycosylphosphatidylinositol lipid anchoring of plant proteins. Sensitive prediction from sequence- and genome-wide studies for Arabidopsis and rice. Plant Physiol.

[74] Rice P, Longden I, Bleasby A (2000). EMBOSS: the European molecular biology open software suite. Trends Genet.

[75] Katoh K, Misawa K, Kuma K, Miyata T (2002). MAFFT: a novel method for rapid multiple sequence alignment based on fast Fourier transform. Nucleic Acids Res.

[76] Stamatakis A, Hoover P, Rougemont J (2008). A rapid bootstrap algorithm for the RAxML web servers. Syst Biol.

