# Supplementary figures and images for: Inter-genome comparison of the Quorn fungus Fusarium venenatum and the closely related plant infecting pathogen Fusarium graminearum
Source: BMC Genomics. 2018 Apr 19;19:269. doi: 10.1186/s12864-018-4612-2 (PMC5907747; doi:10.1186/s12864-018-4612-2)

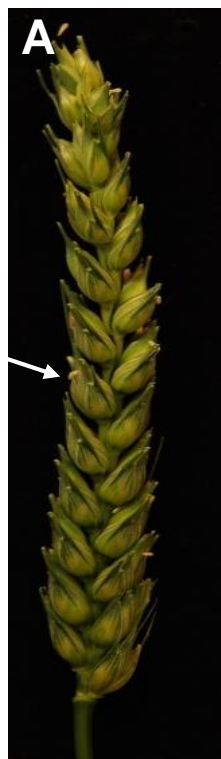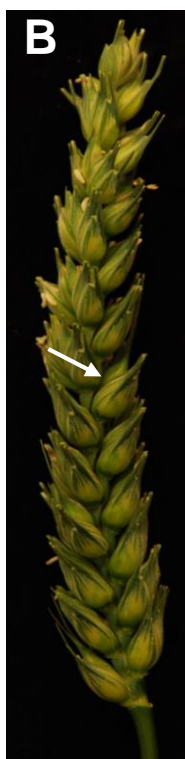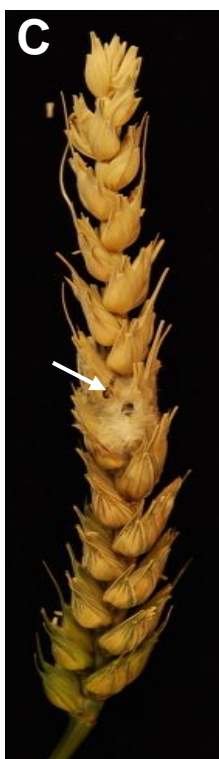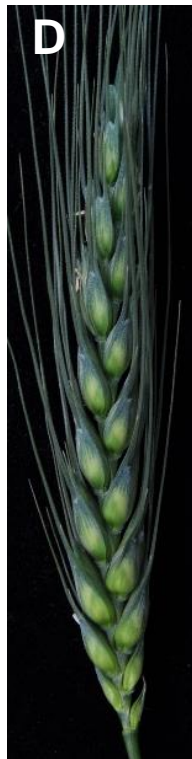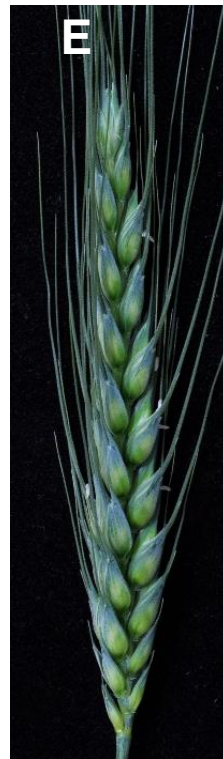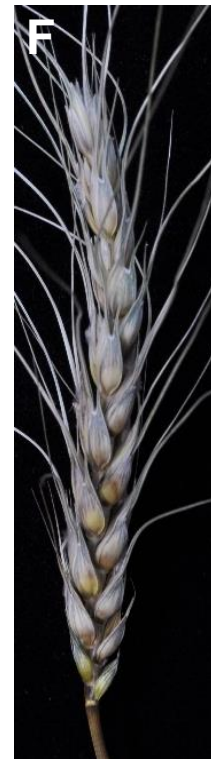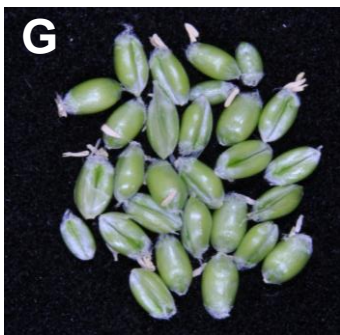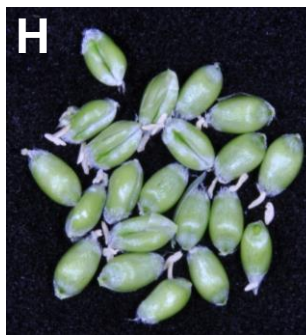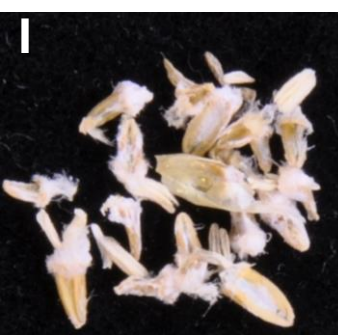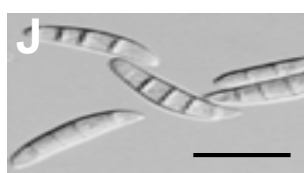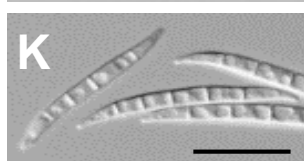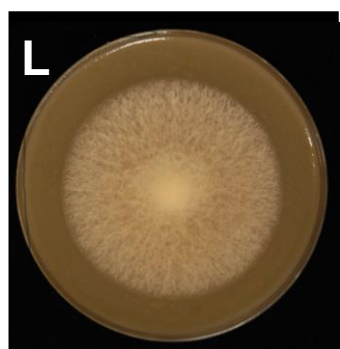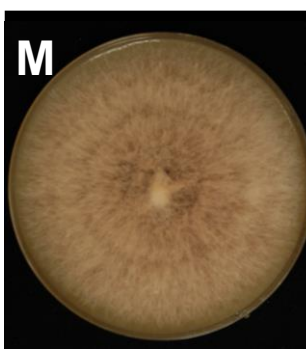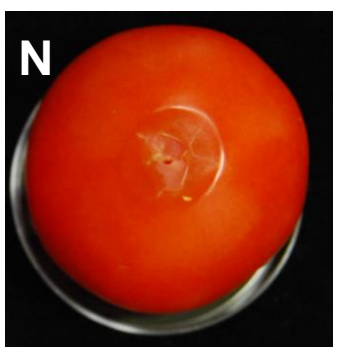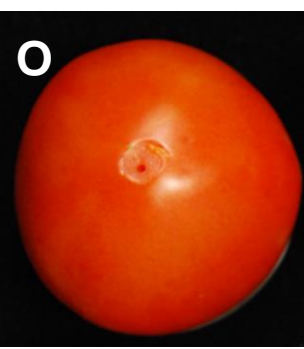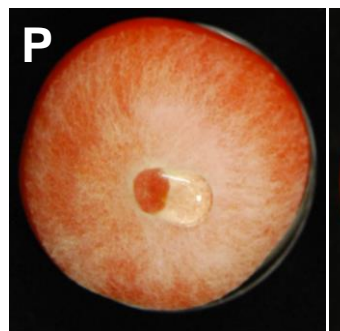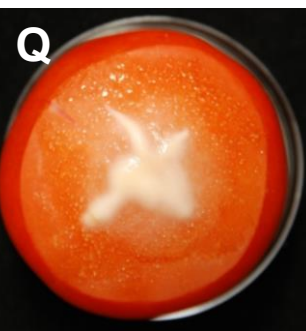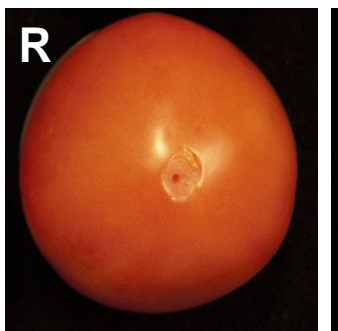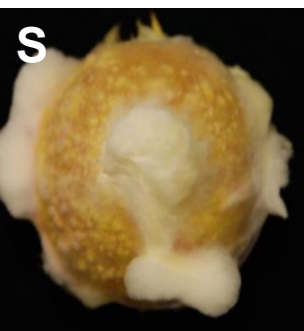

Supplement: Supplementary file 1 — Biological growth characteristics of F. venenatum compared to its close pathogenic relative F. graminearum. (A) Appearance of inoculated wheat head of cv. Bobwhite, 21 days after plug inoculations (dpi) with F. venenatum (Fv), (B) mock inoculation with water, (C) with F. graminearum (Fg). The white arrow indicates the inoculation points. (D) Appearance of wheat head 16 days after spray inoculation (dsi) with Fv, (E) mock spray inoculation with water, (F) spray inoculation with Fg. (G) Wheat seeds collected from a typical spray inoculated wheat head at 21 dsi with Fv, (H) mock spray with water, (I) with Fg. (J) Fv macrospores, (K) Fg macrospores. (L) Fv and (M) Fg growth on PDB plate at 6 days. (N) Fv inoculated tomato fruit at 4 days, (O) water control, (P) Fg. (Q) Fv inoculated tomato fruit at 12 days, (R) water control, (S) Fg. Bars in J, K: 10 μm. (PDF 268 kb) [file 12864_2018_4612_MOESM1_ESM.pdf]

(A)

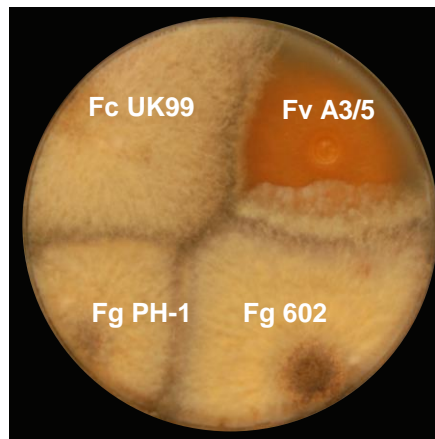

(B)

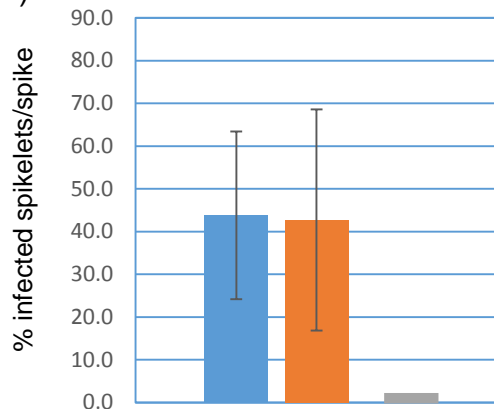

(C)

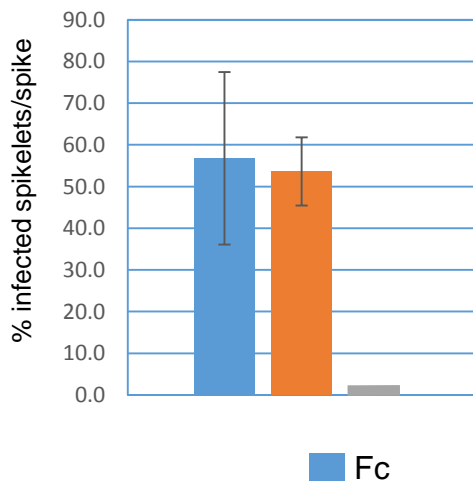

(D)

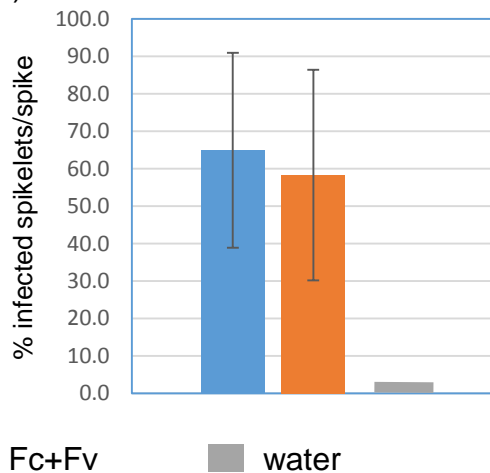

Supplement: Supplementary file 2 — Co-inoculation of F. venenatum with plant-pathogenic Fusaria in vitro and in vivo. (A) Carrot agar plate inoculated with agar plugs of strains Fv A3/5, two isolates of Fg (PH1 and 602), and Fc UK99. A barrage line between strains is visible at day 6. (B) F. culmorum and F. venenatum spores where mixed in equal amounts and co-inoculated into two spikelets at anthesis. As a control, water was inoculated (C) F. venenatum spores were sprayed onto wheat heads at the booting stage to potentially prime plant defence responses, followed by point-inoculation with F. culmorum spores of two heads per spike" should be "two spikelets per head at anthesis. (D) Wheat plants were treated as in C. Just after inoculaton with F. culmorum, wheat heads were sprayed once more with F. venenatum spores until run-off. Error bars show standard deviation, n = 6. (PDF 111 kb) [file 12864_2018_4612_MOESM2_ESM.pdf]

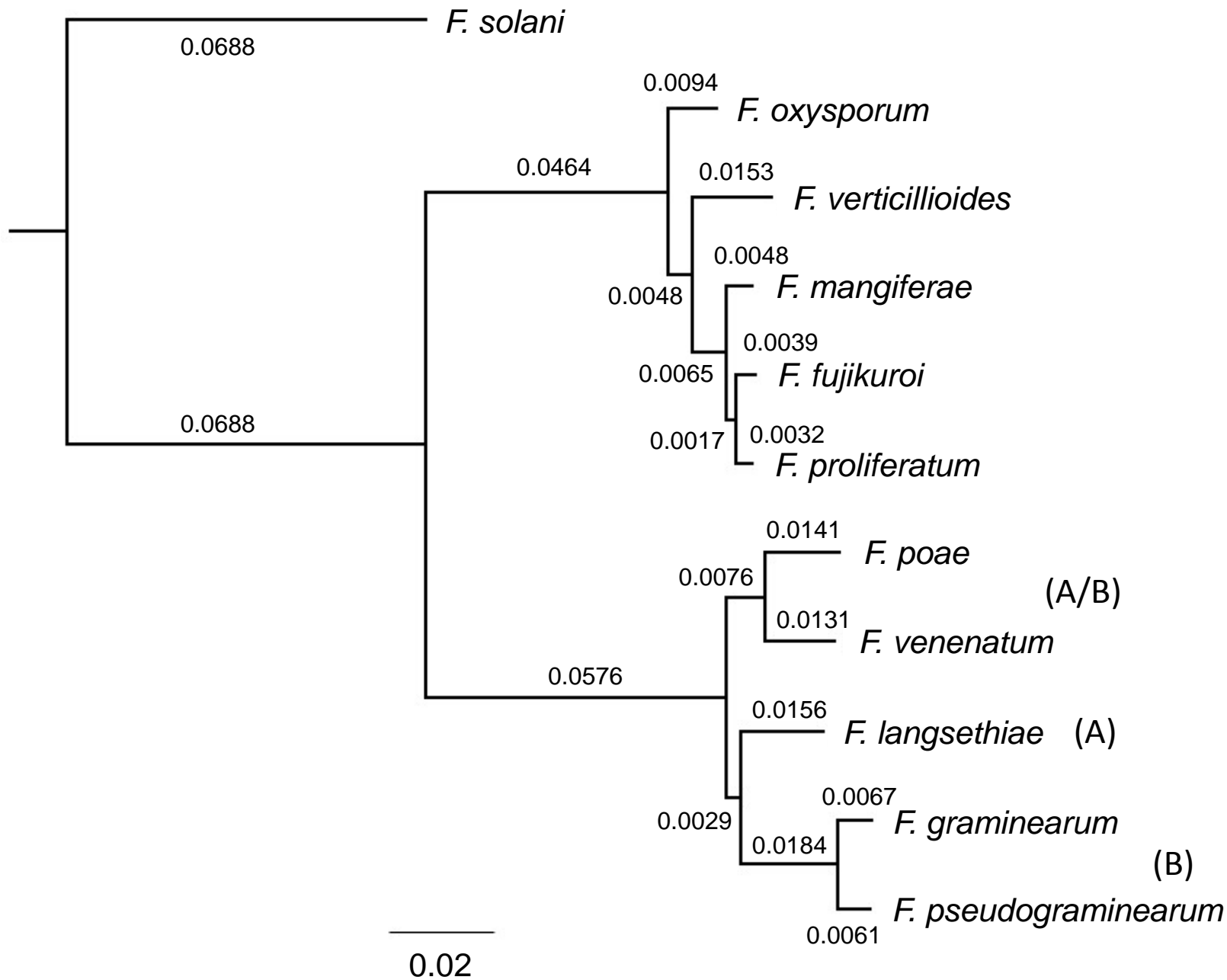

Supplement: Supplementary file 4 — A phylogenetic tree of 904 identified common proteins (see Additional file 3) using F. solani, F. oxysporum, F. verticillioides, F. mangiferae, F. fujikuroi, F. proliferatum, F. poae, F. venenatum, F. langsethiae, F. graminearum and F. pseudograminearum. The substitution rate is on each branch and 100% branch support was found from 100 bootstraps. Type A, B, or A/B trichothecene producers were labelled. The tree was rooted to F. solani. (PDF 104 kb) [file 12864_2018_4612_MOESM4_ESM.pdf]

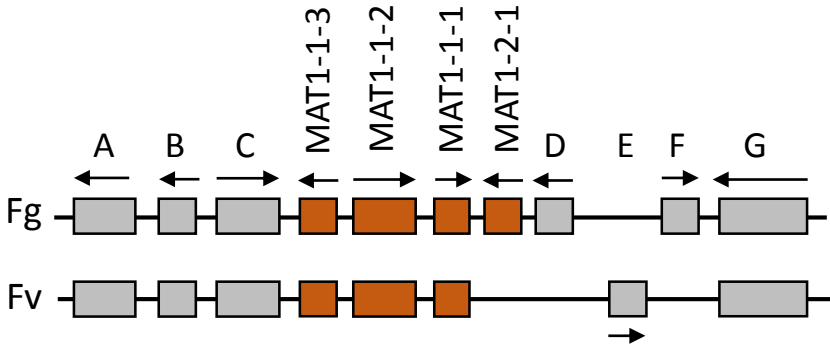

Supplement: Supplementary file 11 — The mating locus within the F. venenatum and F. graminearum genomes. F. venenatum A3/5 contains only the MAT1–1 locus confirming heterothallism and the requirement for a complementary sexual partner. Whilst homothallic F. graminearum possesses both the MAT1–1 and MAT1–2 loci and hence does not require a complementary partner for sexual reproduction. Gene designations: A) FGRRES_08887, B) FGRRES_08888_M, C) FGRRES_08889_M, D) FGRRES_08894, E) FVRRES_05564, F) FGRRES_15525, G) FGRRES_13273_M. (PDF 90 kb) [file 12864_2018_4612_MOESM11_ESM.pdf]

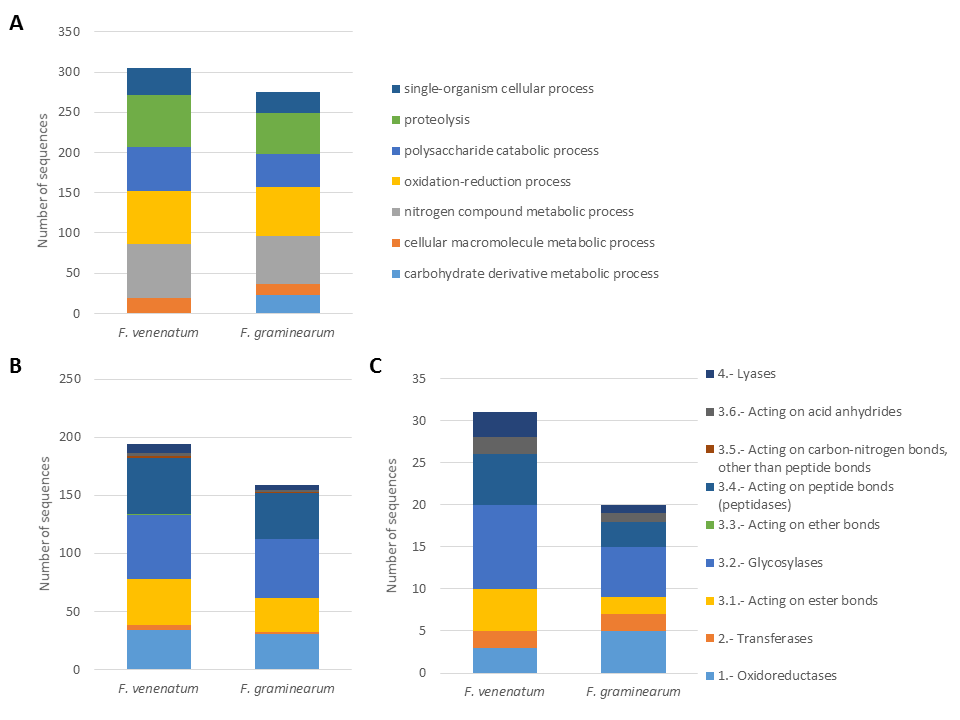

Supplement: Supplementary file 19 — Annotation of Fusarium venenatum and Fusarium graminearum secretomes (A) reveals striking similar functional profiles. The enzymatic repertoire of F. venenatum and F. graminearum including secreted enzymes (B) and species-specific secreted enzymes (C). (TIFF 83 kb) [file 12864_2018_4612_MOESM19_ESM.tif]
