# Supplementary material for: Inter-genome comparison of the Quorn fungus Fusarium venenatum and the closely related plant infecting pathogen Fusarium graminearum
Source: BMC Genomics. 2018 Apr 19;19:269. doi: 10.1186/s12864-018-4612-2 (PMC5907747; doi:10.1186/s12864-018-4612-2)
Supplement: Supplementary file 13 — A figure locating the positions of Fusarium venenatum species-specific genes versus NCBI, in relation to sequences predicted to be the secretome, tRNA, and transposons. (PDF 144 kb) [file 12864_2018_4612_MOESM13_ESM.pdf]

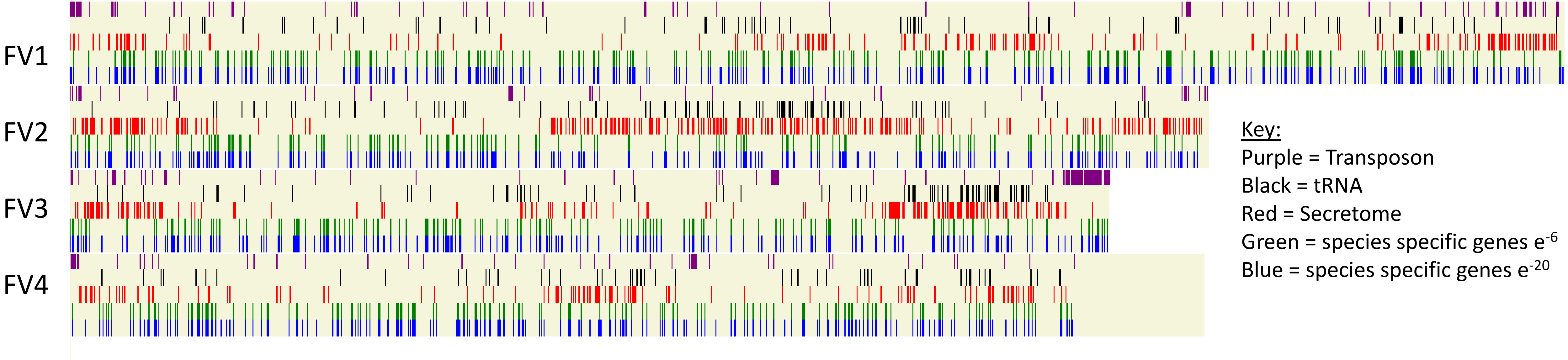
FV1
FV2
FV3
FV4
Key:
Purple = Transposon
Black = tRNA
Red = Secretome
Green = species specific genes e-6
Blue = species specific genes e-20
